# *Getah virus* non-structural protein 2 inhibits type I interferon production by preventing K63-linked polyubiquitination of IKKε and causing widespread cellular shutoff

**DOI:** 10.1128/spectrum.03419-25

**Published:** 2026-06-04

**Authors:** Min Zhao, Likai Ji, Yi Zhao, Shixing Yang, Hongfeng Yang, Bangshun He, Shukui Wang, Wen Zhang

**Affiliations:** 1 Department of Laboratory Medicine, Nanjing First Hospital, Nanjing Medical University 12461, Nanjing, Jiangsu, China; 2 Institute of Critical Care Medicine, The Affiliated People’s Hospital, Jiangsu University 191612, Zhenjiang, China; 3 Department of Laboratory Medicine, School of Medicine, Jiangsu University 12676, Zhenjiang, Jiangsu, China; Shandong First Medical University, Jinan, Shandong, China

**Keywords:** *Getah virus*, non-structural protein 2, IKKε, shutoff, RLR signaling, ubiquitination

## Abstract

*Getah virus* (GETV) is an emerging mosquito-borne alphavirus that poses a growing threat to animal and public health. However, the molecular mechanisms underlying its pathogenesis and immune evasion remain poorly defined. This study demonstrated that GETV infection suppressed the activation of interferon β (IFN-β) or IFN-stimulated response element after poly(I:C) stimulation. By screening nine viral proteins (non-structural proteins 1–4 [nsP1–4] and C-E3-E2-6K-E1), we discovered that the nsP2 protein of GETV could effectively inhibit the production of IFN-β and IFN-stimulated genes. Subsequently, we discovered that nsP2 disrupted the retinoic acid-inducible gene I-like receptor signaling pathway by causing widespread cellular shutoff and directly targeting inhibitor of kappa-B kinase ε (IKKε). Mechanistically, the inhibitory function of nsP2 is contingent upon the protein’s structural integrity and the presence of proline 717 and nuclear localization signal-related residues. Additionally, we illustrated that nsP2 markedly inhibited TRAF2-induced K63-linked polyubiquitination and phosphorylation of IKKε, which is essential for activating IKKε. Altogether, the present study elucidated a new mechanism in which GETV modulates the host’s antiviral immunity.

**IMPORTANCE:**

*Getah virus* (GETV), a multi-host alphavirus of the *Togaviridae* family, imposes a significant economic burden on the swine industry by causing fever, diarrhea, reproductive disorders in sows, and elevated mortality in newborn piglets. Here, we reveal that GETV non-structural protein 2 antagonizes interferon β (IFN-β) production through a dual mechanism: it induces broad cellular shutoff and directly interacts with inhibitor of kappa-B kinase ε (IKKε) to prevent its activation. This strategy effectively disrupts type I IFN responses by suppressing antiviral gene expression, such as IKKε, IFN-I, and IFN-stimulated genes, and impairing retinoic acid-inducible gene I-like receptor signaling activation. Our findings not only uncover a novel mechanism of GETV immune evasion but also establish the rationale for novel therapeutic targets, suggesting a new avenue for the treatment and intervention of GETV infection.

## INTRODUCTION

*Getah virus* (GETV) belongs to the genus *Alphavirus* of the family *Togaviridae* and was initially reported in *Culex* mosquitoes caught in Malaysia in 1955 (1). The first case of porcine GETV infection in China was recorded in Taiwan in 2002. Since then, over 23 provinces in China have reported discovering GETV strains (2, 3). It is an important emerging virus transmitted by mosquitoes, causing infections in multiple vertebrates, including birds, humans, pigs, horses, monkeys, foxes, cattle, and other mammals (3–8). The infected horses are generally self-limiting and present with skin rashes, hind limb edema, transient pyrexia, and lymph node enlargement, while GETV infection in pigs has been linked to sow reproductive disorders and fetal death (8, 9). There have been no reports of definite pathogenicity of GETV in humans so far. Nevertheless, GETV strains are phylogenetically grouped with Ross River virus (RRV), and they share similar antigens with the human pathogen RRV, which has been associated with sudden outbreaks of epidemic polyarthritis in Australia and Papua New Guinea (10). Meanwhile, seroepidemiologic surveys on humans indicate that some fever patients have significantly higher antibody prevalence against GETV compared to healthy individuals. This suggests that GETV poses an epidemic threat to humans similar to other members belonging to the Semliki Forest group of the *Alphavirus* genus, such as RRV, Mayaro virus, Chikungunya virus (CHIKV), Semliki Forest virus (SFV), and O’nyong-nyong virus (3, 11).

The alphavirus has a single-stranded, positive-sense RNA (11.5–12 kb) genome, with two open reading frames, encoding four non-structural proteins (nsPs: nsP1, nsP2, nsP3, and nsP4) and five structural proteins (capsid protein [C], two major envelope glycoproteins [E1 and E2], and two smaller accessory proteins [E3 and 6K]) (12). Non-structural proteins are required for new viral genomic and subgenomic RNA synthesis, protein modification, and immune evasion (10). The alphavirus nsP1 and nsP3 can cap viral positive-strand RNAs, anchor proteins in the plasma membrane, and initiate viral genome replication, respectively. The nsP2 N-terminal superfamily 1 helicase is mainly composed of an N-terminal domain, two conserved Rec-A-like domains (RecA1 and RecA2 domains), and a short linker (13). The C-terminal region of nsP2 is organized into the protease domain and methyltransferase (MTase)-like (MTL) domain, which is believed not to have MTase activity as a result of the absence of crucial structural elements (14). The protease domain located at the C-terminal region of nsP2 is responsible for processing the early non-structural polyprotein (nsP1234 or nsP123) to generate four mature individual non-structural proteins (nsP1–4) (15). The RNA-dependent RNA polymerase nsP4, the first cleaved product of the polyprotein, together with nsP123, nsP23 precursors, or other individual nsPs, forms replication complexes (the early form nsP123-P4, the intermediate form nsP1-P23-P4, and the mature form nsP1-P2-P3-P4) that participate in the viral replication process (16, 17). The capsid protein translated by subgenomic RNA is required for packaging the viral genome RNA and forming an icosahedral nucleocapsid (18, 19). The E2 glycoprotein of alphaviruses has been reported to be associated with cell tropism, host range, and pathogenicity (20). Each functional envelope (E) glycoprotein on the surface of the alphavirus virions contains trimers of E2-E1 heterodimers that are essential for the viral particles’ internalization into host cells through interaction with the entry receptor (21, 22). E3 (the furin-like protease cleavage product of precursor p62) and 6K are small proteins that serve together as the signal sequence to translocate the E2-E1 heterodimers to the endoplasmic reticulum (10, 23).

The crucial function of interferons (IFNs) in restricting virus replication in vertebrates by triggering the expression of numerous IFN-stimulated genes (ISGs) and the retinoic acid-inducible gene I (RIG-I)-like receptor (RLR) pathway plays a key role in the early responses to RNA virus infections (24, 25). Alphavirus infection, like many other RNA viruses, generates substantial amounts of double-stranded RNAs (dsRNAs), acting as pathogen-associated molecular patterns that can be recognized by RLRs in the cytoplasm (26, 27). As the cytosolic receptors RIG-I and melanoma differentiation-associated gene 5 (MDA5) are activated, they in turn recruit mitochondrial antiviral signaling (MAVS, also known as VISA/IPS-1) protein through interactions between their caspase activation and recruitment domains. MAVS then activates downstream signaling of two noncanonical IKKs or IKK-related kinases, TANK-binding kinase 1 (TBK1)/inhibitor of kappa-B kinase ε (IKKε, also known as IKBKE and IKKi) via tumor necrosis factor (TNF) receptor-associated factors (TRAFs) in both IKKγ (also known as NEMO) dependent and independent manner (28). TBK1 and IKKε have been confirmed to be conjugated with the K63-linked polyubiquitin chains, and this modification, synthesized through TRAFs, is required for their activation (29–31). Both kinases share an N-terminal kinase domain (KD), a helical scaffold/dimerization domain (SDD), and a C-terminal domain. The two functional units, KD and SDD, are connected through a ubiquitin-like domain (31). The activated kinases TBK1 and IKKε induce the phosphorylation of IFN regulatory factor 3 (IRF3) and IRF7. Subsequently, phosphorylated IRF3 and IRF7 dimerize, translocate into the nucleus, and trigger transcription of IFN and other proinflammatory cytokines. The activated IRF3 predominantly mediates early, rapid initial immune responses to viral infections. This will induce low levels of IFN-I protein (IFN-β and IFN-α4) expression, whereas IRF7 participates in the robust IFN-α/β gene expression via downstream signaling cascades during the latter stages of viral infection (32–34).

Current research on pathogenic mechanisms is primarily focused on a few well-known members, such as CHIKV, RRV, SFV, Sindbis virus (SINV), and Venezuelan equine encephalitis virus (VEEV) (35–40). However, the strategies utilized by the emerging alphavirus GETV to antagonize host innate immune responses remain poorly understood. Generally, the global shutoff of host cell transcription and protein synthesis is believed to be responsible for alphaviruses evading the antiviral effects of IFNs (40, 41). Previous work has demonstrated that nsP2 of Old World alphaviruses CHIKV, SINV, and SFV rapidly inhibits host gene expression by inducing polyubiquitination and degradation of RPB1, a subunit of the RNA polymerase II (RNAPII) complex that catalyzes the polymerase reaction during cellular RNA transcription (15, 42). The mutation of the NLS within nsP2 retains the protein in the cytoplasm, leading to a decrease in viral genomic RNA synthesis and the loss of the ability to trigger host cell transcriptional shutdown and apoptosis (15, 18). Interestingly, VEEV, CHIKV, SINV, and related alphaviruses also employ other strategies to block IFN-stimulated response element (ISRE)-mediated expression of ISGs, which is controlled by the JAK/STAT signaling pathway, independently of the general host cell shutoff (16, 40). The nsP2 of the atypical Salmonid alphavirus contains two NLS clusters in the C-terminal domain that can block the RIG-I pathway without causing cellular shutoff during infection (24). In addition, Webb et al. (43) identified that CHIKV capsid protein can induce cyclic GMP-AMP synthase (cGAS) degradation via autophagy, thus resulting in a significant inhibition of cGAS-Stimulator of Interferon Gene-dependent IFN-I promoter activation.

Here, we investigated the interaction between GETV and host antiviral responses. It is not surprising that GETV has the ability to inhibit IFN-I production, analogous to other alphaviruses. The results indicated that nsP2 of GETV strongly hindered RLR-induced signaling cascade and antiviral responses via directly targeting the kinase IKKε. We employed different mutated forms of GETV nsP2 (nsP2^KR648/649AA^, nsP2^P717S^, and nsP2^KR648/649AA-P717S^) to verify that GETV nsP2-mediated cellular shutoff can inhibit both the transcription and translation of IKKε. We also investigated the impact of the P717S (proline to serine) mutation on nsP2’s ability to antagonize TRAF2-catalyzed K63-linked polyubiquitination of IKKε, uncovering a new method used by alphavirus nsP2 to subvert the cellular antiviral defenses. These findings will deepen our understanding of the function of alphavirus nsP2 and direct us to develop new therapeutic targets against GETV infection.

## RESULTS

### GETV nsP2 inhibits IFN-β and ISG expression

**Fig 1:**
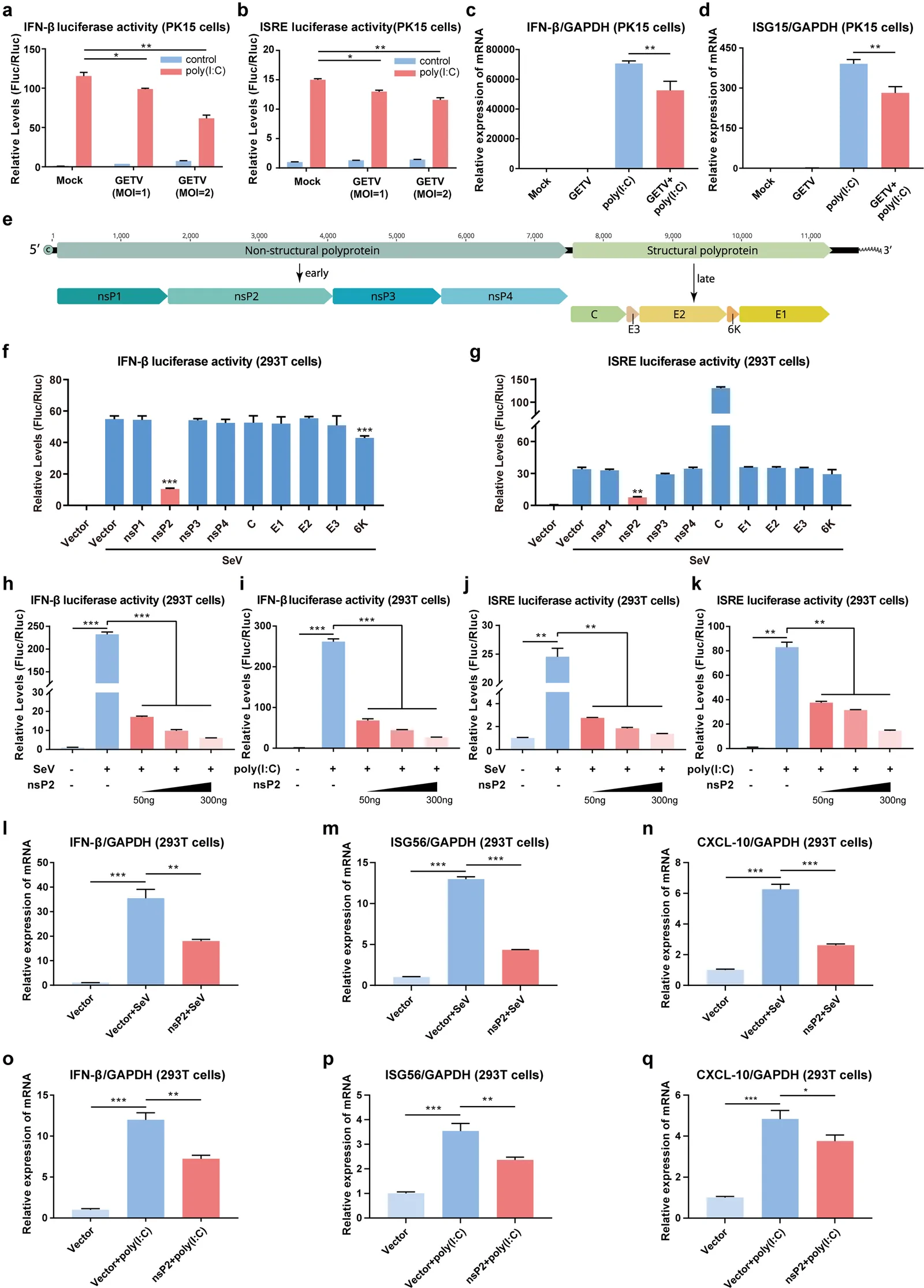
GETV nsP2 protein attenuates type I IFN production. (**a and b**) PK15 cells were transfected with the pGL3-IFN-β-Luc (**a**) or pGL3-ISRE-Luc (**b**) reporter plasmid together with pRL-TK-Luc control plasmid and then mock-infected or infected with GETV at an MOI of 0.5 or 1 for 24 h. At 12 h post-infection, cells were transfected with poly(I:C) or left untreated for an additional 12 h, and supernatants of cells were then harvested for dual-luciferase reporter assays. The data are presented as the fold induction of the IFN-β and ISRE promoter activity. (**c and d**) PK15 cells were mock-infected or infected with GETV at an MOI of 1. At 12 hpi, cells were transfected with or without poly(I:C) for an additional 12 h. Relative mRNA levels of IFN-β (**c**) and ISG15 (**d**) gene expression were analyzed by qRT-PCR. (**e**) The schematic diagram of the GETV genomic architecture. (**f and g**) Effect of nine GETV proteins on Sendai virus (SeV)-induced IFN-β (**f**) or ISRE (**g**) promoter activation. HEK293T cells were transfected with plasmids expressing the indicated viral proteins, along with the pGL3-IFN-β-Luc or pGL3-ISRE-Luc and pRL-TK-Luc plasmids. After 24 h, cells were left uninfected or infected with SeV for another 12 h. (**h–k**) HEK293T cells were transfected with increasing amounts of nsP2-expressing plasmids or empty vector, along with pGL3-IFN-β-Luc (**h and i**) or pGL3-ISRE-Luc (**j and k**) and pRL-TK-Luc plasmids. After 24 h, the cells were infected (or not) with SeV (**h and j**) or transfected (or not) with poly(I:C) (**i and k**) for an additional 12 h, and then luciferase activities were measured. (l**–q**) Quantitative PCR analysis of IFN-β (**l and o**), ISG56 (**m and p**), and CXCL-10 (**n and q**) mRNA levels. HEK293T cells were transfected with the nsP2 expression plasmid or the empty vector. After 24 h, the cells were stimulated (or not) by SeV (**l–n**) or transfected (or not) with poly(I:C) (**o–q**) for an additional 12 h. GAPDH was used as an internal control gene for normalization. All results are shown as the means ± SD of three independent experiments, two-tailed Student’s *t* test. *, *P* < 0.05; **, *P* < 0.01; and ***, *P* < 0.001. MOI, multiplicity of infection.

The vital function of IFNs in response to viral infection has driven the development of multiple mechanisms in many alphaviruses to counteract or evade the host antiviral IFN response, facilitating the rapid spread of viral infections (39). As a member of the *Alphavirus* genus, we hypothesized that GETV might inhibit the expression of IFNs to evade the innate immune response. To investigate whether GETV infection inhibits IFN-β production and signaling, we measured IFN-β and ISRE promoter activities using dual-luciferase reporter assays and quantified IFN-β and ISG mRNA levels by quantitative real-time reverse transcription PCR (qRT-PCR), respectively. Considering that PK15 (porcine kidney-15) cells are more susceptible to GETV infection compared to human-derived cell lines such as HEK293T (human embryonic kidney) and U251 (human glioblastoma) cells (2), we used PK15 cells to study the IFN antagonistic activity after GETV infection. Western blotting results showed that GETV could replicate in PK15 cells (Fig. S1a). As shown in Fig. 1a and b, GETV infection significantly inhibited poly(I:C)-induced IFN-β and ISRE promoter activation in a dose-responsive manner in PK15 cells. When analyzed by qRT-PCR, we also found that GETV infection hindered the expression of porcine IFN-β and IFN-stimulated gene 15 (ISG15) at the mRNA level after poly(I:C) treatment (Fig. 1c and d), consistent with the results obtained by dual-luciferase reporter assays. These results suggest that GETV infection interrupts poly(I:C)-induced IFN-β and ISG production, blunting the host antiviral immune responses.

To further explore which proteins of GETV could negatively regulate the innate immune responses, we cloned all GETV genes, including non-structural genes nsP1–4 and structural genes C, E3, E2, 6K, and E1 (Fig. 1e). Western blot showed that the nine genes could be successfully expressed in HEK293T cells (Fig. S1b). To screen the GETV expression library, we then performed dual luciferase assays to measure the human IFN-β and ISRE promoter-driven luciferase activity in mock-infected or Sendai virus (SeV)-infected cells (Fig. 1f and g). We identified that nsP2 had the most obvious inhibitory effect on the IFN-β induction (Fig. 1f). The regulatory effect of nsP2 on ISRE promoter activation was similar in SeV-infected HEK293T cells (Fig. 1g). Thus, we focused on the multifunctional protein nsP2, the main regulator of the alphavirus life cycle, which is important for viral genome replication and subversion of antiviral defense in host cells (14). Overexpression of nsP2 remarkably inhibited the SeV/poly(I:C)-triggered promoter activities of IFN-β and ISRE in a dose-dependent manner in HEK293T cells (Fig. 1h through k). Moreover, RT-qPCR results indicated that SeV/poly(I:C) stimulation induced the production of endogenous transcripts of IFN-β, ISG56, and cytokine CXC-chemokine ligand 10 (CXCL-10). The mRNA levels of these human antiviral genes were significantly reduced in nsP2-overexpressing cells (Fig. 1l through q). In addition, endogenous IFN expression levels in HEK293T cells transfected or mock-transfected with GETV nsP2 were evaluated using an IFN bioassay with IFN-sensitive vesicular stomatitis virus (VSV)-expressing green fluorescent protein (VSV-GFP). The GFP expression was analyzed by fluorescence microscopy, and the titers of the released VSV-GFP progeny virus were measured by the TCID_50_ assay. The data showed that GETV nsP2 strongly restored the poly(I:C)-restricted replication of VSV-GFP (Fig. S1c and d). Under the same conditions, nsP2 also suppressed the activities of IFN-β and ISRE promoters stimulated by poly(I:C) in PK15 cells (Fig. S1e and f) and effectively inhibited the transcription of porcine IFN-β, ISG15, and CXCL-10 mRNA (Fig. S1g through i). In contrast, GETV nsP2 expression did not reduce the nuclear factor κB (NF-κB) promoter activity in a dose-dependent manner (Fig. S1j), nor did it significantly affect the mRNA levels of the NF-κB-associated cytokines TNF-α and IL-1β in SeV-infected cells (Fig. S1k and l). These results showed that GETV nsP2 inhibits IFN-β production and signaling in an NF-κB-independent manner, following stimulation with SeV or poly(I:C).

### GETV nsP2 impairs IFN-β activation by affecting the RLR signaling pathway

**Fig 2:**
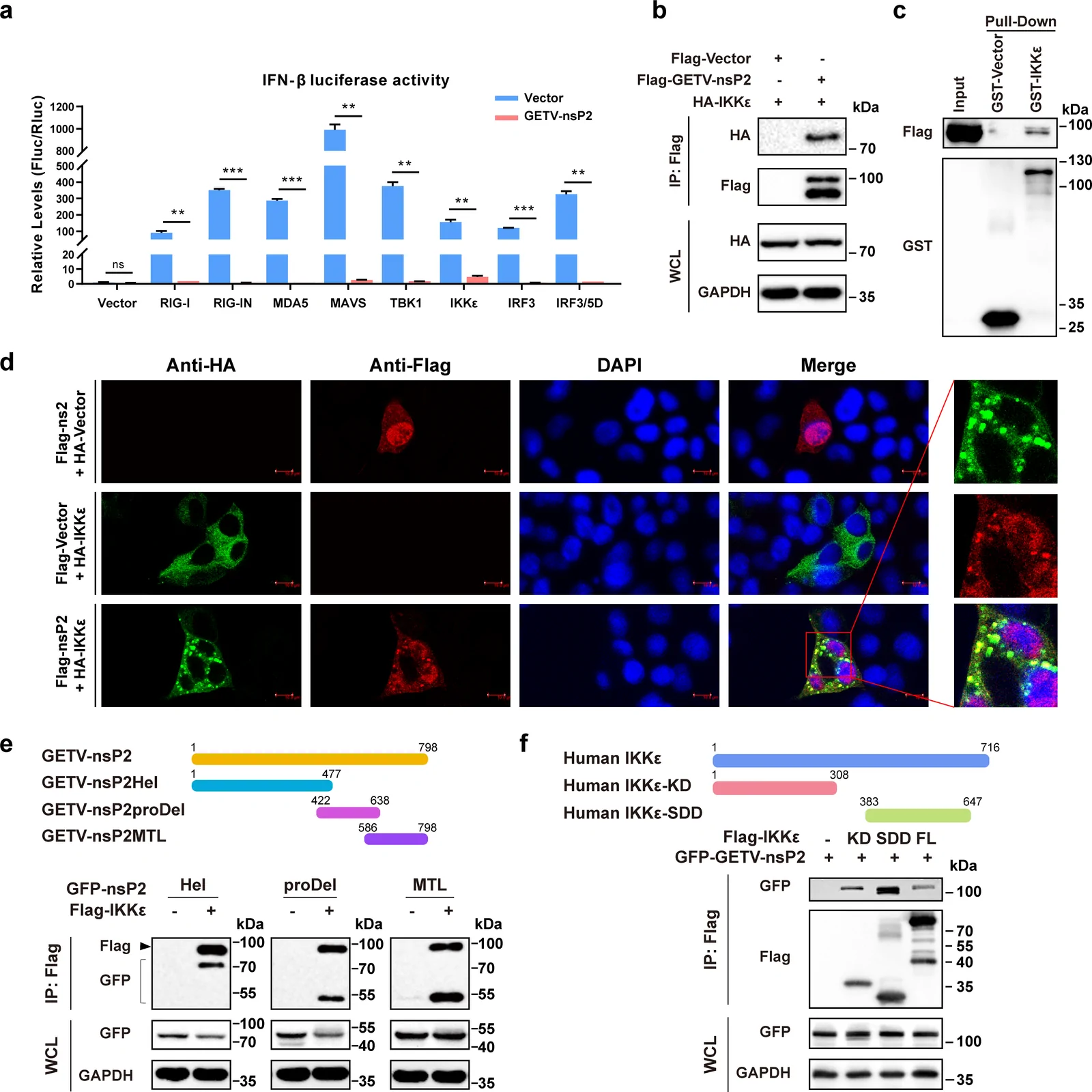
GETV nsP2 protein targets and directly binds to IKKε. (**a**) HEK293T cells were co-transfected with pGL3-IFN-β-Luc, pRL-TK-Luc, and plasmids expressing RIG-I, RIG-IN, MDA5, MAVS, TBK1, IKKε, IRF3, or IRF3-5D, with or without the nsP2 plasmid for 24 h. Data represent means ± SD of triplicate samples. **, *P* < 0.01; ***, *P* < 0.001; ns, non-significant differences in data upon Student’s *t* test (two-tailed). (**b**) Co-immunoprecipitation and western blotting analysis of HEK293T cells transfected with HA-IKKε expression plasmid together with the Flag-tagged nsP2 or empty vector for 36 h. The immunocomplexes were precipitated by incubation with Flag-affinity gel, and the eluted proteins were explored using western blotting assays. (**c**) The glutathione S-transferase (GST) fusion IKKε or GST protein was expressed in *Escherichia coli* BL21 (DE3) and subsequently purified by GST glutathione agarose resin. HEK293T cells were transfected with Flag-nsP2 for 36 h, and supernatants of the whole cell extracts were harvested. The interaction between the eukaryotic protein Flag-nsP2 and purified prokaryotic expression proteins was analyzed by a GST pull-down experiment. GST-tagged empty vector served as a negative control. (**d**) HeLa cells were transfected with Flag-nsP2 or HA-IKKε or co-transfected with both and processed 36 h later for an indirect immunofluorescence assay with the indicated antibodies. Scale bar, 10 μm. (**e**) Top panel: schematic representation of nsP2 and three derived truncated constructs. Hel, helicase domain (1–477 aa); proDel, protease domain (422–638 aa); and MTL, methyltransferase-like domain (586–798 aa). Bottom panel: HEK293T cells were co-transfected with plasmids expressing GFP-tagged nsP2 truncated mutant proteins along with or without Flag-IKKε and processed 36 h later for co-immunoprecipitation and western blotting analysis. (**f**) Top panel: schematic illustration of human IKKε and two derived mutants. KD, kinase domain (1–308 aa); SDD, helical scaffold/dimerization domain (383–647 aa). Bottom panel: HEK293T cells were co-transfected with plasmids encoding Flag-tagged IKKε full-length or truncated mutant proteins along with or without GFP-nsP2 and processed 36 h later for co-immunoprecipitation and western blotting analysis. All data shown are representative of two or more independent experiments.

Because SeV and poly(I:C) are important inducers of the RLR-mediated IFN-β signaling pathway, we speculated that nsP2 could target one or several molecules of the RLR signaling pathway to block the activation of host innate immune responses. Dual-luciferase reporter assays showed that transfection with RIG-IN (the constitutively active N-terminal helicase domain of RIG-I) or MDA5 notably triggered the activation of the IFN-β promoter in HEK293T cells, but the enhanced activation was significantly lower in the presence of GETV nsP2 (Fig. S2a and b). The qRT-PCR results indicated that the expression of endogenous human IFN-β, ISG56, and CXCL-10 mRNA induced by RIG-IN was remarkably decreased in the presence of GETV nsP2 (Fig. S2c through e). We next investigated at what level in the pathway nsP2 suppressed the production of IFN-β using dual-luciferase reporter assays. HEK293T cells were co-transfected with empty vector or nsP2 expression plasmid and a variety of plasmids expressing key signaling molecules involved in the RLR pathway, including RIG-I, RIG-IN, MDA5, MAVS, TBK1, IKKε, IRF3, and IRF3-5D (the constitutively active form of IRF3) together with reporter plasmids. Results showed that all the tested key components mediated a 90- to 990-fold activation of the IFN-β promoter, whereas overexpression of nsP2 displayed a general inhibitory effect on RLR-induced IFN-β signaling (Fig. 2a). All the data suggested that GETV nsP2 could interfere with the production of IFN-β by antagonizing RLR signaling.

### Association and colocalization of GETV nsP2 with IKKε

**Fig 3:**
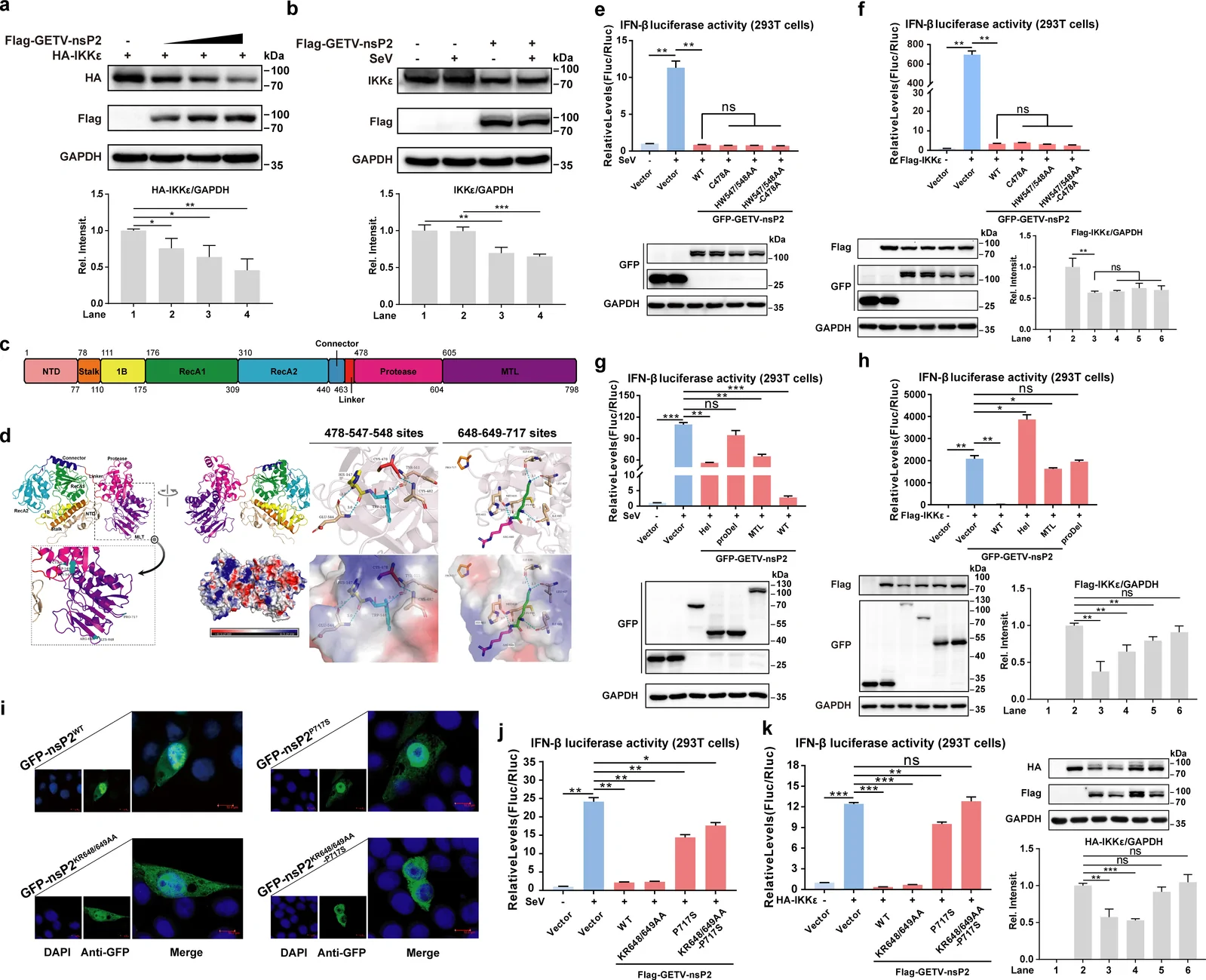
GETV nsP2 downregulates IKKε protein and mRNA expression. (**a**) HEK293T cells were co-transfected with plasmids encoding HA-tagged IKKε and elevated doses of Flag-tagged nsP2 for 24 h. (**b**) HEK293T cells were transfected with Flag-tagged nsP2 or empty vector plasmid for 24 h and then mock-infected or infected with SeV for 12 h. Protein abundances of IKKε or GAPDH (loading control) were analyzed by western blotting. (**c**) Schematic illustration of the domain arrangement of nsP2 protein. (**d**) The predicted tertiary structural model of nsP2 protein. (**e and f**) Interferon β luciferase activity of HEK293T cells stimulated by SeV (**e**) or Flag-IKKε (**f**) was analyzed by a dual-luciferase reporter assay with GFP-tagged wild-type nsP2 or its protease-deficient mutants (nsP2^C478A^, nsP2^HW547/548AA^, and nsP2^C478A-HW547/548AA^). (**g and h**) Interferon β luciferase activity of HEK293T cells stimulated by SeV (**g**) or Flag-IKKε (**h**) was analyzed by a dual-luciferase reporter assay with GFP-tagged wild-type nsP2 or its truncated constructs (Hel, proDel, MTL, and MTL^P717S^). (**i**) Subcellular localization of the wild-type nsP2 or the indicated mutants (nsP2^KR648/649AA^, nsP2^P717S^, and nsP2^KR648/649AA-P717S^) in HeLa cells was determined by IFA. (**j and k**) Interferon β luciferase activity of HEK293T cells stimulated by SeV (**j**) or HA-IKKε (**k**) was analyzed by a dual-luciferase reporter assay with Flag-tagged wild-type nsP2 or its mutants (nsP2^KR648/649AA^, nsP2^P717S^, and nsP2^KR648/649AA-P717S^). All data shown are representative of three independent experiments. Data represent means ± SD per group. Significance was calculated using Student’s *t* test (two-tailed). *, *P* < 0.05; **, *P* < 0.01; ***, *P* < 0.001; and ns, non-significant.

Based on the existing evidence of the interaction between GETV nsP2 and TBK1 (44) and given the functional similarity between IKKε and TBK1, we hypothesize that nsP2 similarly targets IKKε to broadly suppress interferon signaling. To identify whether GETV nsP2 interacts with IKKε, we conducted co-immunoprecipitation (Co-IP) and western blotting experiments. The result showed that GETV nsP2 could bind with IKKε (Fig. 2b). To further examine whether GETV nsP2 and IKKε directly interact, we performed a glutathione S-transferase (GST) pull-down assay. As shown in Fig. 2c, the pull-down assay using purified GST fusion proteins confirmed the direct binding of IKKε to nsP2. Similar to other Old World alphavirus nsP2 (16, 45, 46), the GETV nsP2 protein is distributed in both the cytoplasm and the nucleus, with a predominant localization in the nucleus (Fig. 2d and 3i). Furthermore, intracellular interaction between nsP2 (red) and IKKε (green) was validated by the indirect immunofluorescence assay (IFA), as the overlapping fluorescent signal (yellow) was clearly distributed in the cytoplasm of PK15 cells (data not shown) and HeLa (human cervix carcinoma) cells (Fig. 2d). Overall, our findings unveil that nsP2 of GETV specifically and directly interacts with IKKε in the cytoplasm.

We further addressed which domains of GETV nsP2 are essential for its interaction with IKKε. Based on the crystal structure and functional domain analysis of CHIKV nsP2 (13), we constructed three truncated mutants of GETV nsP2: Hel (1–477 aa), proDel (422–638 aa), and MTL (586–798 aa). Next, we tested their interactions with full-length Flag-tagged IKKε or empty vector in HEK293T cells. Co-IP results showed that the nsP2 truncated mutant containing the MTL domain was significantly precipitated by the IKKε protein (Fig. 2e). IKKε shares two crucial domains, N-terminal kinase domain (1–308 aa) and C-terminal helical scaffold/dimerization domain (383–647 aa), which are responsible for binding to IRF3/7 and TRAFs, respectively (28, 47). We then investigated which domains of IKKε are required for the interaction with GETV nsP2. As depicted in Fig. 2f, the SDD domain of IKKε, not the KD domain, was the main region retaining its interaction with nsP2. Our results further confirmed the interaction between GETV nsP2 and IKKε.

### GETV nsP2 downregulates the expression of IKKε

We next dissected how GETV nsP2 suppresses IKKε-mediated IFN-β activation. To determine whether nsP2 could decrease the expression of IKKε, we co-transfected HA-tagged IKKε and Flag-tagged GETV-nsP2 expression plasmids or the empty vector into HEK293T cells, and then the cells were harvested and subjected to western blotting analysis. As seen in Fig. 3a, the protein levels of exogenous IKKε were gradually downregulated, accompanied by an increased amount of GETV nsP2. To further clarify whether nsP2 could reduce the endogenous IKKε, we transfected the Flag-GETV-nsP2 plasmid or the empty vector into cells, followed by mock infection or infection with SeV for 12 h. The western blotting results showed that GETV nsP2 could arrest endogenous expression of IKKε, in comparison to the empty vector, regardless of SeV infection (Fig. 3b). These results suggested that overexpression of GETV nsP2 could inhibit the protein synthesis of both exogenous and endogenous IKKε.

### The protease activity of GETV nsP2 is dispensable for inhibition of IFN-β activation and IKKε expression

The GETV nsP2 protein was predicted to have three main functional regions: a superfamily 1 helicase domain, a protease domain, and a methyltransferase-like domain (Fig. 3c). To test whether GETV nsP2 utilizes its proteolytic processing activity to cleave host protein, we predicted three conserved catalytic residues (C478, H547, and W548) in the protease region via multiple comparisons (48–50) and then constructed three enzyme-deficient mutants (nsP2^C478A^, nsP2^HW547/548AA^, and nsP2^C478A-HW547/548AA^) (Fig. 3d; Fig. S3a and b). Compared to the wild-type nsP2, these enzymatically inactive mutants still had the same capacity to abrogate the IFN-β promoter activation mediated by SeV and IKKε (Fig. 3e and f). There was no apparent difference in the protein expression level of exogenous IKKε in cells expressing wild-type or mutated GETV nsP2 plasmid (Fig. 3f). Our results indicate that the GETV nsP2-associated inhibitory effect on IFN-β promoter activation and IKKε protein expression is independent of its protease activity.

### The structural integrity of the nsP2 protein is crucial for its inhibitory effect

To define which domains of nsP2 are involved in inhibiting SeV/IKKε-mediated IFN-β production, we performed a dual luciferase reporter assay. As depicted in Fig. 3g, the Hel domain of nsP2 showed the highest ability to suppress SeV-induced IFN-β activity in HEK293T cells, followed by the MTL domain. However, when Hel and MTL plasmids were expressed, the inhibitory effect of GETV nsP2 was partially retained but still greatly diminished compared to that of wild-type nsP2, indicating that the inhibitory function of nsP2 depends on the protein’s structural integrity. The proDel truncated mutant alone had almost no inhibitory effect on SeV-induced IFN-β promoter activation (Fig. 3g), which is consistent with our previous findings that the GETV nsP2-associated inhibitory effect on IFN-β promoter activation is independent of its protease activity. We also investigated the effect of the tested truncations on IKKε-induced IFN-β activity or IKKε expression. Compared to the empty vector control, both Hel and MTL truncations remarkably decreased the expression of IKKε, while only the MTL mutant modestly impaired IKKε-induced IFN-β activity (Fig. 3h). Surprisingly, the Hel domain significantly upregulated the activity of the IFN-β promoter induced by IKKε. We speculated that the differences in the Hel domain’s regulation of SeV- or IKKε-triggered IFN-β promoter activity may be due to its ability to influence the post-translational modifications of IKKε.

### GETV nsP2-induced transcriptional and translational shutoff contributes to the inhibition of IKKε expression

During infection, typical alphaviruses induce transcriptional cellular shutoff, cytopathic effects, and even cell death (18). *Getah virus* is classified into the Old World arthritogenic alphavirus subgroup, which typically employs nsP2 for host cell transcription and translation inhibition (15, 51). Several studies have revealed that the conserved proline (P) residue and NLS-related residues located in the C-terminus of Old World alphavirus nsP2, such as P718 and K649/R650 in CHIKV nsP2, play key roles in alphavirus-induced host cell shutoff (15, 16, 19). Therefore, we have to question whether the lower protein abundance of IKKε is a consequence of GETV nsP2-induced cellular transcription and protein synthesis shutoff. The sequence homology analysis of the C-terminal region of nsP2 highlighted the presence of a highly conserved proline residue at position 717 of GETV nsP2, along with the putative NLS-related sequence composed of residues lysine (K) 648 and arginine (R) 649 (Fig. S3a). Guided by the predicted secondary and tertiary structural data (Fig. 3d; Fig. S3a), we mutated the three conserved and surface-exposed residues (P717S and K648A/R649A) to better elucidate whether these residues within the C-terminal domain are necessary for suppressing cellular gene transcription and expression. We first monitored the subcellular localization of the three nsP2 mutants via immunofluorescence assays in HeLa cells (Fig. 3i). Both the wild-type nsP2 and the mutated GETV-nsP2^P717S^ were abundantly found in the nucleus. Different from the findings obtained for NLS mutations in nsP2 of CHIKV and SFV (16, 18), the GETV-nsP2^KR648/649AA^ variant remained present in the nucleus of transfected cells, whereas the combination of substitutions P717S, K648A, and R649A in nsP2 contributed to an exclusively cytoplasmic variant (Fig. 3i). HEK293T cells were transfected with a pEGFP-N1 vector alone or together with plasmids expressing wild-type or mutated GETV nsP2 to further confirm the essential role of these residues (P717, K648, and R649) in modulating GETV nsP2-mediated host cell shutoff activity. We evaluated the mRNA and protein expression levels of the enhanced green fluorescent protein (EGFP) by qRT-PCR and western blotting assays, respectively. Overexpression of wild-type nsP2 produced a marked decrease in both EGFP transcript and protein levels, and the wild-type GETV nsP2 dose-dependently suppressed EGFP protein expression (Fig. S3c through e). Similar to the wild-type nsP2, the GETV-nsP2^KR648/649AA^ mutant caused a strong reduction in mRNA abundance and protein expression of EGFP. However, GETV-nsP2^KR648/649AA-P717S^ and GETV-nsP2^P717S^ mutants had no significant effect on EGFP mRNA or protein levels (Fig. S3c, d, and f), indicating that these residues within the C-terminal region of nsP2 are indeed involved in regulating its shutoff activity.

Then, we sought to determine whether there is a difference in the ability of the wild-type and three GETV nsP2 mutants to inhibit IFN-β production and IKKε mRNA or protein expression. As shown in Fig. 3j and k, the shutoff-deficient mutant GETV-nsP2^P717S^ and the NLS-deficient mutant GETV-nsP2^KR648/649AA-P717S^ strongly abrogate nsP2’s ability to block the activation of the IFN-β promoter stimulated by SeV or IKKε, but the GETV-nsP2^KR648/649AA^ mutant behaved similarly to wild-type nsP2 in its ability to inhibit the IFN-β promoter activation. GETV-nsP2^KR648/649AA^ mutant led to shutoff of both the IKKε protein and mRNA to reduced levels similar to the wild-type nsP2 (Fig. 3k; Fig. S3g). Not surprisingly, GETV-nsP2^P717S^ and GETV-nsP2^KR648/649AA-P717S^ mutants did not inhibit the transcript and protein levels of IKKε in the absence of host shutoff activity. Western blotting analysis with anti-Flag antibody showed higher levels of protein accumulation for GETV-nsP2^P717S^ and GETV-nsP2^KR648/649AA-P717S^, compared to the low levels for the wild-type nsP2 and GETV-nsP2^KR648/649AA^ mutant (Fig. 3k; Fig. S3d). This suggests that mutations at positions P717 and K648/R649, or P717 alone, do not suppress the expression of the GETV nsP2 gene. Together, these data indicate that GETV nsP2 reduces both the mRNA and protein synthesis of IKKε, dependent on the global host cell transcriptional and translational shutoff induced by nsP2.

### GETV nsP2 inhibits the activation of IKKε

**Fig 4:**
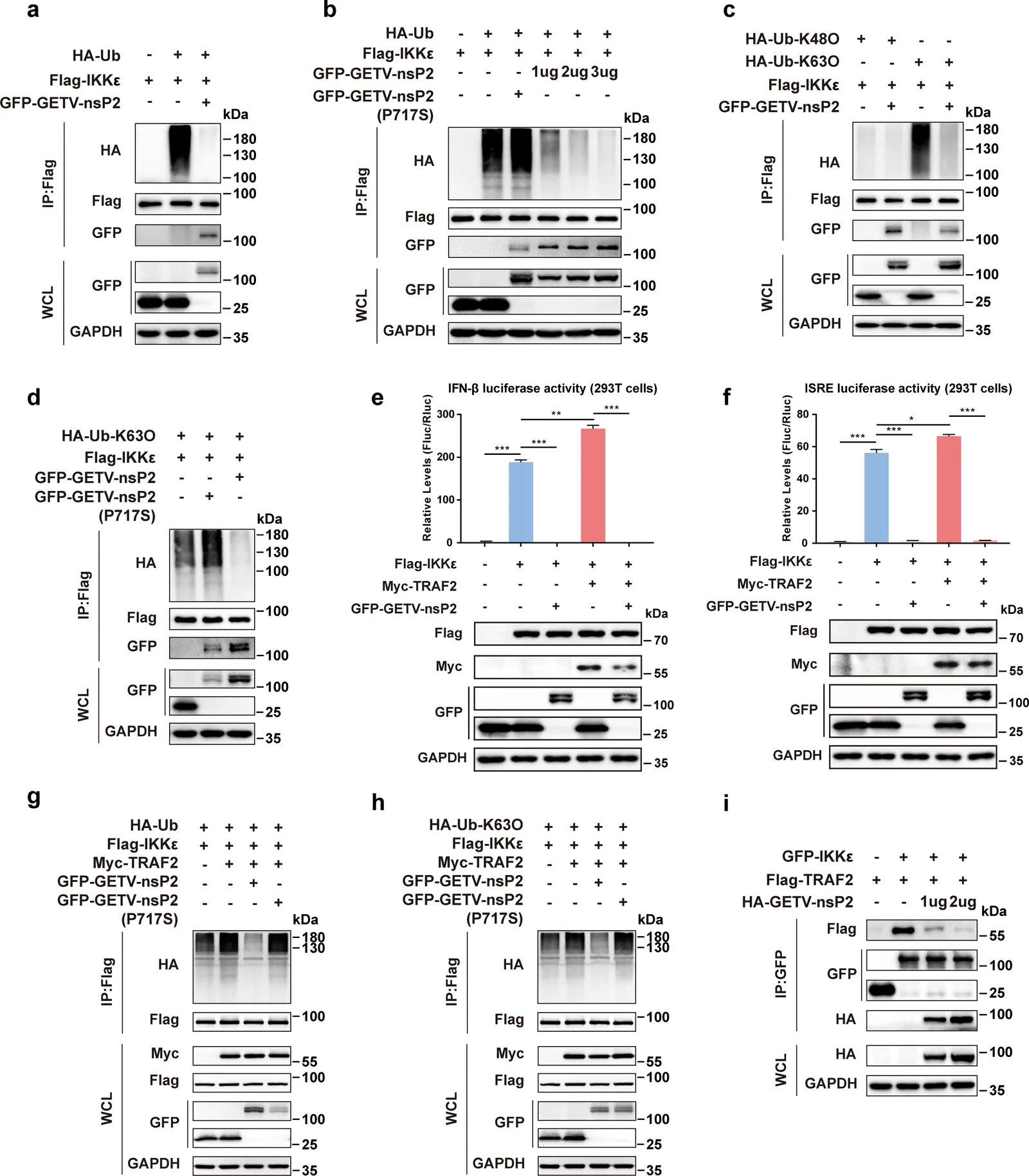
GETV nsP2 decreases TRAF2-induced K63-linked polyubiquitination of IKKε. (**a**) HEK293T cells were co-transfected with Flag-IKKε, GFP-nsP2, and wild-type HA-tagged ubiquitin expression plasmids. (**b**) HEK293T cells were co-transfected with Flag-IKKε, wild-type HA-Ub, and dose-dependent, GFP-tagged wild-type nsP2 plasmids or its mutant nsP2^P717S^. (**c**) Plasmids encoding Flag-IKKε, GFP-nsP2, and HA-tagged ubiquitin K48 only or K63 only were co-transfected into HEK293T cells. (**d**) HEK293T cells were co-transfected with plasmids expressing HA-Ub-K63O, Flag-tagged IKKε, and GFP-tagged wild-type nsP2 or its mutant nsP2^P717S^. After 36 h, cell lysates were subjected to co-immunoprecipitation with Flag-affinity gel, followed by western blotting analysis. (**e and f**) HEK293T cells were co-transfected with plasmids expressing pGL3-IFN-β-Luc (**e**) or pGL3-ISRE-Luc (**f**), pRL-TK-Luc, and Flag-IKKε, with or without TRAF2 and nsP2, with the empty vector plasmid as the control. Dual-luciferase reporter assays and western blotting were performed after 24 h. Data represent means ± SD per group. *, *P* < 0.05; **, *P* < 0.01; and ***, *P* < 0.001. (**g and h**) HEK293T cells were co-transfected with plasmids encoding Flag-IKKε, Myc-TRAF2, and GFP-tagged wild-type nsP2 or its mutant nsP2^P717S^, together with wild-type HA-Ub (**g**) or mutant HA-Ub-K63O plasmid (**h**). After 32 h, cell lysates were subjected to co-immunoprecipitation with Flag-affinity gel, followed by western blotting to analyze the ubiquitination of IKKε. (**i**) HEK293T cells were co-transfected with GFP-IKKε, Flag-TRAF2, and HA-nsP2 plasmids. After 32 h of transfection, cells were lysed for co-immunoprecipitation by GFP-affinity gel. All data shown are representative of two or more independent experiments.

Protein ubiquitination is the process of adding ubiquitin chains or ubiquitin-like molecules to substrate proteins, and it is involved in nearly all aspects of eukaryotic biology. Two main lysine residues of ubiquitin, K48 and K63, are well known for their distinctive functions in mammalian cells (52). It operates as a versatile post-translational modification and plays an indispensable role in modulating the activity of host proteins, such as IKKε (28, 30). To determine the effects of nsP2 on IKKε ubiquitination, the IKKε plasmid was co-transfected with HA-tagged ubiquitin and GFP-tagged GETV-nsP2 plasmids or an empty vector in HEK293T cells. As seen in Fig. 4a and b, IKKε is robustly ubiquitinated by ubiquitin, while the ubiquitination of IKKε was greatly decreased to the background level in the presence of GETV nsP2. Next, we found that the mutation of P717S could abolish the deubiquitination function of IKKε by nsP2 (Fig. 4b). To further clarify which type of polyubiquitination linkage in IKKε could be decreased by nsP2, an IKKε expression plasmid was co-transfected with GFP-tagged GETV nsP2 or empty plasmid together with HA-tagged ubiquitin K48 only or K63 only (HA-Ub-K48O or HA-Ub-K63O) mutated plasmid, which retains only a single lysine residue (K48 or K63, respectively). IKKε has been reported to be specifically modified by K63-linked ubiquitin chains at lysines 30 and 401, which are essential for IKKε function in immune signaling pathways (30). Here, we also observed that IKKε was ubiquitinated by K63-linked, instead of K48-linked, polyubiquitin chains, and overexpression of GETV nsP2 specifically deubiquitinated the K63-linked polyubiquitination of IKKε (Fig. 4c). In contrast, the GETV-nsP2^P717S^ mutated plasmid did not decrease the K63-linked polyubiquitination of IKKε (Fig. 4d). The results above prove that the P717 site of GETV nsP2 is pivotal for removing K63-linked polyubiquitin chains from IKKε.

It is known that activation of IKKε depends not only on K63-linked polyubiquitination but also on phosphorylation at Ser172 (S172) and Thr501 (T501) (30, 53, 54). Therefore, we further examined whether nsP2 affects the two phosphorylation statuses of IKKε. The results showed that the protein levels of phosphorylated IKKε at S172 and T501 were both reduced in nsP2-overexpressing cells, suggesting that nsP2 can inhibit IKKε activation by regulating its phosphorylation levels (Fig. S4a and b). Given prior evidence that nsP2 does not influence TBK1 phosphorylation (44), we subsequently assessed whether nsP2 affects K63-linked polyubiquitination of TBK1. As expected, overexpression of nsP2 did not markedly suppress TBK1 K63-linked polyubiquitination (Fig. S4c). In summary, nsP2 modulates IKKε activation by influencing its K63-linked polyubiquitination and phosphorylation, but not TBK1 activation.

The E3 ligase TRAF2 can catalyze K63-linked ubiquitination of IKKε (28, 30). To assess the impact of TRAF2 on IFN-I induction and signaling induced by IKKε, we conducted dual-luciferase reporter assays. We discovered that the transactivation activity of IKKε was further elevated following co-transfection with TRAF2 plasmid. The synergistic activation of IFN-β or ISRE promoter induced by IKKε coupled with TRAF2 was significantly impeded by GETV nsP2 (Fig. 4e and f). Since TRAF2 can catalyze the K63-linked polyubiquitination of IKKε, we further delineated whether GETV nsP2 could affect the TRAF2-induced ubiquitination of IKKε. Consistent with the luciferase-based reporter assays, overexpression of TRAF2 plasmid markedly enhanced IKKε ubiquitination and K63-linked polyubiquitination in HEK293T cells (Fig. 4g and h). Subsequently, we observed that the wild-type GETV nsP2 could remarkably reduce the TRAF2-enhanced ubiquitination and K63-linked polyubiquitination of IKKε, whereas the mutated nsP2^717S^ did not (Fig. 4g and h). To elucidate the influence of GETV nsP2 on the IKKε/TRAF2 interaction, the Co-IP assay was performed. As shown in Fig. 4i, increasing amounts of GETV nsP2 led to a strong reduction of IKKε/TRAF2 association. Further analysis revealed that GETV nsP2 could also coprecipitate with TRAF2 (Fig. S4d). Collectively, our results indicated that GETV nsP2 significantly hinders the interaction between the E3 ubiquitin ligase TRAF2 and IKKε, thereby inhibiting K63-linked polyubiquitination of IKKε and impairing IKKε-mediated signaling and subsequent induction of IFN-I.

## DISCUSSION

With the expanding epidemic range and host range of GETV, this neglected alphavirus has brought continuous economic losses in many regions and is considered a potential threat to public health. GETV can cause pathological damage to many vertebrates, represented by pigs and horses (3). Shi et al. (6) found that GETV exhibited a broad tissue tropism and severe pathogenicity in the infected blue fox individuals. In experimental mouse models of GETV infection, clinical symptoms were observed, including respiratory distress, weight loss, mobility impairment, testicular damage, and even death (55, 56). In a recent study, we also detected high viral titers of GETV infection in the heart tissues of dead red pandas (57). The cytopathic effects of GETV have also been visible not only in mosquito cells but also in HEK293T, PK15, Vero (African green monkey), BHK-21 (baby hamster kidney), and HmLu-1 (hamster lung) cells (2, 58). Altogether, GETV can readily replicate and disseminate in various hosts with strong immune systems, leading to pathological changes in the infected cells and tissues. Li et al. (59) have discovered that IFN-I (IFN-α/ω) and IFN-III (IFN-λ3) pre-treatments failed to suppress GETV early replication in swine testis cells, suggesting the capacity of GETV to escape IFN-I/III responses. Nonetheless, there is still a lack of enough attention on GETV, and the studies on the virus-host interactions, including antiviral response strategies of host cells and the innate immune escape mechanisms of this virus, are still insufficient. Here, we show that IFN-I production and signaling could be suppressed during GETV infection, thereby leading to immune evasion and persistent infection of GETV in vertebrate cells.

Type I interferon response is the first line of the host’s defense against alphavirus infection. Given the vital role of the IFN-I system in repressing virus infection, many alphaviruses have developed specific mechanisms to modulate IFN-I production in order to circumvent the host’s antiviral immunity (60). Apart from non-structural protein 2, envelope glycoproteins (E1 and E2) of CHIKV and small membrane protein (TF) of SINV are also known to antagonize host type I interferon responses (42, 61). However, how GETV antagonizes the host innate immunity, such as the IFN-I system, remains obscure. Given that nsP2 functioned as a strong antagonist of IFN-β production, we focused our subsequent investigations on this protein and did not further explore the role of other GETV proteins in immune evasion (Fig. 1f). The dsRNA replicative intermediates of alphaviruses can be sensed by Toll-like receptor 3 and the cytoplasmic RNA virus sensors RIG-I and MDA5 (26). The members of the RLR family interact with downstream adaptor proteins to activate signaling cascades, which ultimately result in the expression of IFN-I and inflammatory chemokines. To determine whether GETV nsP2 downregulates the RLR-mediated type-I interferon response, we evaluated its effect on signaling pathways triggered by RIG-I and MDA5, as well as their downstream molecules—MAVS, TBK1, IKKε, and IRF3—in the induction of IFN-I. Our study indicates that overexpression of nsP2 in HEK293T cells can broadly suppress IFN production activated by these components (Fig. 2a). Recent research has shown that GETV nsP2 combines with TBK1 and effectively inhibits the activation of IRF3 (44). In addition to TBK1, IKKε is also an essential kinase for the IRF3 signaling pathway. Therefore, we initially focused on the regulatory relationship between GETV nsP2 and IKKε, revealing a direct interaction between nsP2 and IKKε (Fig. 2b through f). However, the way in which nsP2 targets IKKε to inhibit IKKε-triggered signaling cascades is yet to be elucidated.

Based on a previous study by Law et al. (13), the multifunctional GETV nsP2 was predicted to consist of an N-terminal domain, a helicase core domain (Stalk-1B-RecA1/2), a protease domain, and a C-terminal S-adenosylgfp-L-methionine-dependent RNA methyltransferase-like domain (Fig. 3c). Co-IP experiments revealed that the MTL domain (586–798 aa) was the main region retaining the interaction with nsP2 (Fig. 2f). We found that nsP2 prevented IKKε protein expression in a dose-dependent manner (Fig. 3a). The protease activity of nsP2 is critical for viral genome amplification during alphavirus replication because it is the main protease responsible for cleaving the non-structural polyprotein (62). In this study, nsP2^C478A^, nsP2^HW547/548AA^, and nsP2^C478A-HW547/548AA^, the mutations at the protease active sites of nsP2, had no effect on the activation of IFN-β promoter stimulated by SeV or IKKε, as well as the protein expression of IKKε (Fig. 3e and f), suggesting the protease activity of nsP2 is dispensable for its function in the RLR signaling pathway. The C-terminal MTL domain of Old World alphaviruses nsP2 was found to have no methyltransferase activity, but it is well-known to be associated with the global inhibition of host cell protein transcription and translation induced by nsP2 (15, 19). We identified that only the MTL mutant modestly impaired IKKε-induced IFN-β activity, and it could also affect the expression of IKKε (Fig. 3h). Therefore, it is of interest to understand whether the C-terminal MTL domain of GETV nsP2 plays a key role in the general inhibition of host gene expression, similar to other Old World alphaviruses.

Our findings demonstrated that inactivating the host shutoff function of nsP2 through point mutations in the P717 or NLS sites disrupted the viral protein’s ability to hinder cellular transcription and protein synthesis (Fig. S3c through f). Similar to other Old World alphaviruses (16, 45, 46), GETV nsP2 is localized in both the cytoplasm and the nucleus (Fig. 2d and 3i). The transcription and translation inhibitory activity of the Old World alphavirus nsP2 strictly depends on its accumulation in the nucleus (15). In contrast to the mutation of NLS in the CHIKV nsP2^KR649/650AA^, double mutations of K648A/R649A in nsP2 rarely prevented nsP2 translocating to the nucleus, leading to minimal impact on the antagonism of SeV/IKKε-mediated IFN-β promoter activation (Fig. 3i through k). In contrast, introducing the point mutations at three sites (KR648/649AA-P717S) in its C-terminal domain prevented nsP2 from translocating to the nucleus (Fig. 3i), thereby reversing the shutoff of host protein induced by the viral protein. Hence, the GETV nsP2^KR648/649AA-P717S^ mutant not only lost its nuclear functions but also lacked host shutoff activity. The integrity of the N-terminal helicase domain and the C-terminal MTL domain of Old World alphavirus nsP2 is crucial for counteracting the IFN-β response (15). Therefore, we examined the truncated mutants of GETV nsP2 on IFN-β induction and IKKε expression. Data demonstrated that cells co-transfected with the full-length nsP2 plasmid showed a greater reduction in IFN-β activity and IKKε protein expression compared to cells co-expressing Hel or MTL truncated plasmids (Fig. 3g and h), indicating that the host shutoff mechanism utilized by nsP2 requires its protein’s structural integrity. Interestingly, compared to the full-length nsP2, the helicase region alone enhanced IKKε-induced IFN-β activity (Fig. 3h), but it was still involved in negatively regulating SeV-mediated IFN-β promoter activation and IKKε protein expression (Fig. 3g and h). This suggests that there may be another regulatory mechanism employed by GETV nsP2 specifically targeting IKKε.

Apart from causing host cell shutoff, these types of RNA viruses, such as influenza viruses and coronaviruses, have evolved other strategies to confront or circumvent the IFN system, for example, by hijacking cellular proteins or by regulating the ubiquitination of host proteins (63–65). Ubiquitination is one of the most important and pervasive modified modes of protein that plays critical roles in the signal transduction of the RLR signaling pathway (66, 67). Generally, K63-linked ubiquitination marks proteins for signal transduction, leading to non-proteolytic changes in protein function, whereas K48-linked ubiquitin chains tag proteins for proteasomal degradation in mammalian cells (68). In this light, we investigated whether GETV nsP2 specifically regulates the ubiquitination level of IKKε and observed that nsP2 significantly inhibited the polyubiquitination of IKKε (Fig. 4a and b). We then elucidated which type of polyubiquitination was affected by nsP2. Through co-transfecting nsP2 and ubiquitin HA-Ub-K48O or HA-Ub-K63O mutated expression plasmids, we discovered that nsP2 specifically targeted and reduced the K63-linked polyubiquitin chains of IKKε, but not K48-linked ones (Fig. 4c). Akhrymuk et al. (15) reported that the shutoff-deficient mutant (P726G or P726L) of SINV nsP2 could affect proteasome-mediated Rpb1 degradation by preventing the polyubiquitination of Rpb1, suggesting the potential role of this conserved proline of the MTL domain in modulating the ubiquitination of host proteins. As we speculated, overexpression of the nsP2^P717S^ plasmid could subvert and even enhance the ubiquitination and K63-linked polyubiquitination of IKKε in contrast to the wild-type nsP2 (Fig. 4b and d). Ubiquitination is a dynamic and reversible process of post-translational modification, which is balanced by both E3 ligases and deubiquitinases. Previous studies have shown that the E3 ubiquitin ligase TRAF2 (coiled-coil domain) directly interacts with IKKε (SDD domain) and synthesizes K63-linked polyubiquitin chains to modify and activate IKKε involved in signal transduction of the NF-κB pathway (28, 30). Here, we found that the SDD domain of IKKε was the main region retaining the interaction with nsP2 (Fig. 2f). Overexpression of nsP2 reduced TRAF2-catalyzed K63-linked polyubiquitination of IKKε and the interaction of TRAF2 with IKKε (Fig. 4h and i), indicating that nsP2 may compete with TRAF2 for binding to the SDD domain of IKKε to suppress its activation. In addition to modulating IKKε ubiquitination, our study showed that nsP2 also perturbs IKKε phosphorylation (Fig. S4a and b). IKKε is known to contribute not only to IRF3 activation but also to IFN-driven STAT1 signaling, thereby regulating the expression of a distinct subset of ISGs (54, 69, 70). In particular, phosphorylation of IKKε at T501 has been implicated in virus- and IFN-β-induced activation, and activated IKKε can promote STAT1 phosphorylation to expand the antiviral transcriptional responses. Therefore, our finding that nsP2 suppresses IKKε phosphorylation at T501 suggests that nsP2 may attenuate downstream STAT1 activation and consequently compromise IKKε-dependent ISG expression. Altogether, GETV nsP2 effectively antagonizes IFN-I production through two different strategies. Determination of the relative contribution of suppressing IFN-β expression via host shutoff or the direct inhibition of IKKε activation needs further experiments.

**Fig 5:**
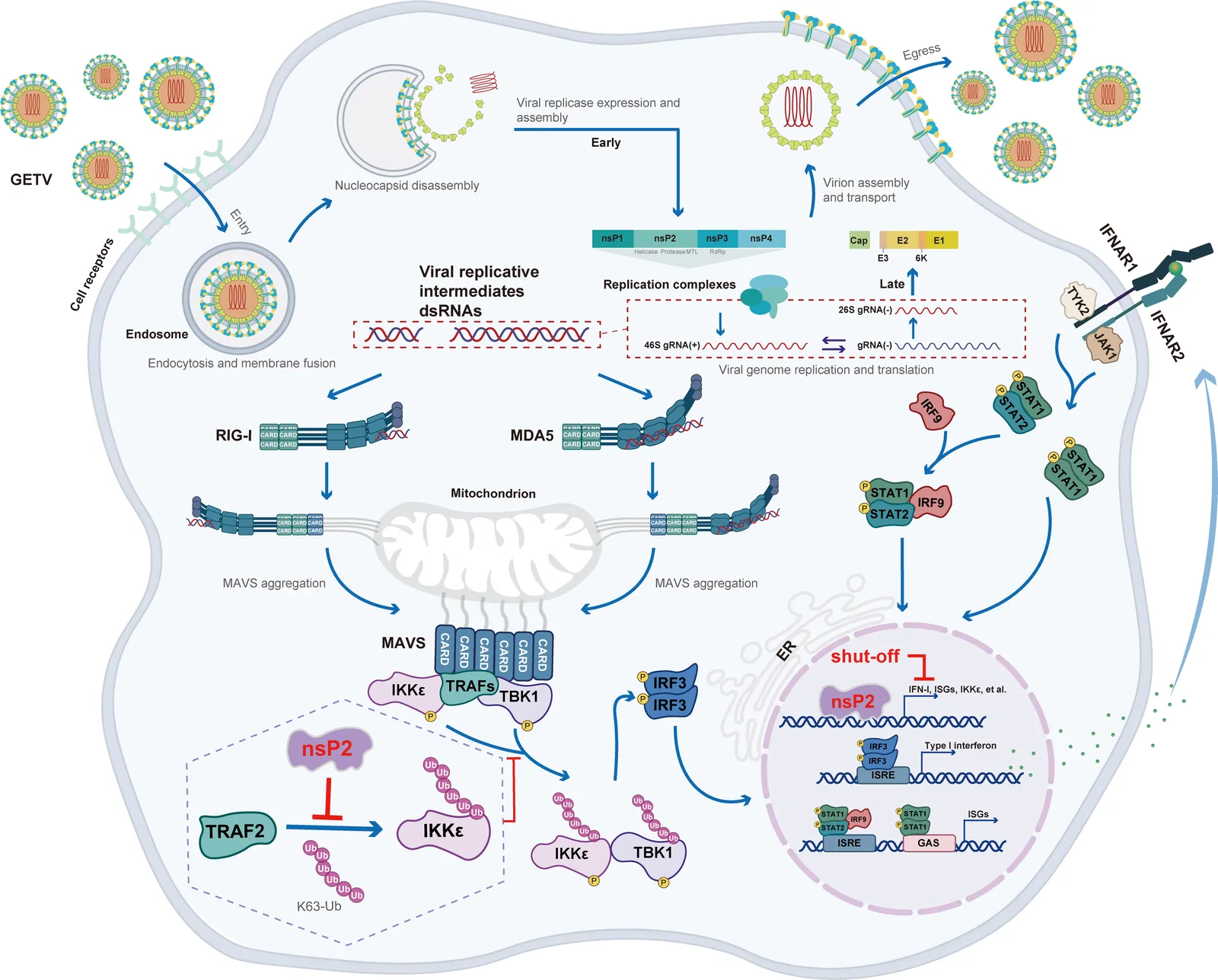
Schematic depicting the negative regulatory role of GETV nsP2 in response to the RLR signaling pathway. During GETV infection and replication, the synthetic viral dsRNA replicative intermediates are recognized by cytosolic recognition receptors, such as RIG-I and MDA5. Upon ligand recognition, RIG-I and MDA5 engage with the mitochondrion-anchored adaptor protein MAVS, which facilitates the activation of downstream IKK-related kinases, TBK1 and IKKε, and subsequent signaling cascades, ultimately resulting in the production of IFN-I. IKKε ubiquitination is essential for host antiviral immunity, and its K63-linked polyubiquitin chains could be catalyzed by the TRAF family member, TRAF2. In the cytoplasm, the GETV nsP2 protein could directly bind to the host protein IKKε. Then, nsP2 removes TRAF2-mediated K63-linked polyubiquitin chains from IKKε to inhibit IKKε-triggered signaling cascades. In the nucleus, GETV nsP2 may bind to DNA through its N-terminal domain and interact with RPB1, a subunit of the RNAPII complex, using its C-terminal region. However, the mechanism of the association needs further in-depth research. This binding initiates global host shutoff, reducing cellular gene expression, such as IKKε, IFN-I, and ISGs. Proline 717 of GETV nsP2 plays a crucial role in both scenarios.

In summary, we demonstrated viral protein nsP2 as a strong negative regulator of GETV in antiviral responses by imposing global host shutoff or directly deubiquitinating K63-linked IKKε (Fig. 5). Overexpression of GETV nsP2 decreased the expression of IFN-β and ISGs triggered by SeV or poly(I:C) in both HEK293T and PK15 cells. It also displayed a general inhibitory effect on molecules in the RLR pathway that activate the IFN-β promoter. The GETV nsP2^P717S^ and nsP2^KR648/649AA-P717S^ mutants, which abolish host shutoff activity, significantly recovered SeV/IKKε-mediated IFN-β production compared to the wild-type nsP2-expressing cells. Besides, GETV nsP2 targeted and directly interacted with IKKε and removed TRAF2-mediated K63-linked polyubiquitin chains from IKKε. Importantly, the P717 residue in the MTL domain of nsP2 was in charge of K63-linked IKKε deubiquitination.

## MATERIALS AND METHODS

### Antibodies and reagents

The rabbit polyclonal antibody (pAb) against GETV E2 protein was kindly provided by Prof. Tongling Shan (Shanghai Veterinary Research Institute, China). Anti-HA (Cat# H9658) and anti-Flag (Cat# F1804) mouse monoclonal antibodies (mAbs) and anti-HA (Cat# H6908) rabbit mAb were from Sigma-Aldrich. Anti-GAPDH rabbit mAbs were from ABclonal (Cat# A19056). Horseradish peroxidase (HRP)-conjugated anti-mouse immunoglobulin (IgG) antibody was purchased from ABclonal (Cat# AS003), whereas HRP-conjugated anti-rabbit IgG antibody was obtained from Cell Signaling Technology (Cat# 7074). Anti-Flag, anti-Myc, anti-IKKε, and anti-phospho-IKKε (S172) rabbit mAbs were from Cell Signaling Technology (Cat# 14793S, 2278, 2905, and 8766, respectively). Anti-GFP rabbit pAb (Cat# 31002ES) was from YEASEN. Anti-GST mouse mAb, Alexa Fluor 488-labeled goat anti-rabbit IgG (H + L) secondary antibody, and Alexa Fluor 555-labeled Donkey anti-mouse IgG (H + L) secondary antibody were from Beyotime (Cat# AF5063, A0423, and A0460, respectively).

### Cell culture and virus

The GETV strain used in this study was kindly gifted by Prof. Tongling Shan (Shanghai Veterinary Research Institute, China). SeV and recombinant VSV-GFP strains were originally preserved in our laboratory. Viral supernatants were titrated and stored at −80°C. HEK293T (Cat# CRL-3216), PK15 (Cat# CCL-33), and HeLa (Cat# CCL-2) cells were purchased from the American Type Culture Collection. These cells were cultured in Dulbecco’s Modified Eagle’s Medium (BBI, Cat# E600003) containing 10% heat-inactivated fetal bovine serum (Sigma-Aldrich, Cat# F8687), 100 U/mL penicillin, and 100 µg/mL streptomycin (BBI, Cat# E607011) at 37°C under 5% CO_2_ humidified atmosphere.

### Plasmids and transfection

The genes encoding GETV non-structural (nsP1–4) and structural proteins (C-E3-E2-6k-E1) were PCR amplified from a tissue sample collected from a red panda infected with GETV (57). Each gene fragment was purified and thereafter cloned into the eukaryotic expression vector p3× FLAG-CMV-14/Amp containing a C-terminal triple Flag tag (3× Flag). The GETV nsP2 gene was also cloned into the pAcGFP1-C1 (with a GFP tag at the N terminus) and the pEGFP-N1 (with a GFP tag at the C terminus) vectors. The 3× Flag or GFP-fused GETV nsP2 plasmids were further used as templates for amplifying six site-directed mutants (C478A, HW547/548AA, C478A-HW547/548AA, KR648/649AA, P717S, and KR648/649AA-P717S) and three truncations (Helicase, proDel, MLT). Human RIG-I, MDA5, MAVS, TBK1, IKKε, and IRF3 genes were amplified from complementary DNA (cDNA) generated from HEK293T cells and cloned into a pcDNA3.0+ vector with a Flag tag at the N-terminus. Human IKKε was also cloned into the plasmid pCMV-HA. Human TRAF2 was cloned into the plasmid pcDNA3.1-Myc. The N-terminal GST-tagged human IKKε fragment was cloned and inserted into BamHI/BamHI sites of the prokaryotic expression pCold-GST vector (Clontech Laboratories, Inc., Cat# 3372). The luciferase reporter plasmids and the *Renilla* luciferase reporter vector (pRL-TK-Luc) have previously been described (71, 72). All plasmids used in this study were verified by DNA sequencing. All primers used for constructing plasmids are listed in Table S1.

### Dual-luciferase reporter assay

Cells were seeded in 24-well plates and transiently co-transfected using Lipo6000 Transfection Reagent (Beyotime, Cat# C0526) with a luciferase reporter plasmid (pGL3-IFN-β-Luc, pGL3-ISRE-Luc, or pGL3-NF-κB-Luc [100 ng/well]) and an internal control plasmid (pRL-TK-Luc [10 ng/well]), together with the indicated expression plasmids or empty vector. At 24 h post-transfection, cells were mock-infected or infected with SeV or left untreated or treated with poly(I:C) HMW (high molecular weight) (APExBIO, Cat# B5551) for an additional 12 h. After 24 or 36 h of transfection, cell lysates were harvested, and firefly luciferase and *Renilla* luciferase activities were determined using a Dual-Luciferase Reporter Assay Kit (Vazyme, Cat# DL101-01). Data are shown as the relative levels of firefly luciferase activity normalized to the *Renilla* luciferase activity.

### Quantitative real-time reverse transcription PCR

Total RNA was extracted from cells with TRIzol Reagent (Invitrogen, Cat# 15596026) and subjected to reverse transcription reactions using reverse transcriptase (Vazyme, Cat# R312-01). The generated first-strand cDNA was used as the template for qPCR with specific primer pairs, universal Blue qPCR SYBR Green Master Mix (YEASEN, Cat# 11184ES08), and ABI 7500 PCR system (USA). GAPDH or 18S rRNA was used as an endogenous control gene for normalization, and all data are expressed as relative fold change. The qRT-PCR primer sequences of target genes are displayed in Table S2.

### Co-immunoprecipitation assay and western blotting analysis

We harvested cells in precooled immunoprecipitation lysis buffer (Beyotime, Cat# P0013) with 1 mM phenylmethylsulfonyl fluoride (Beyotime, Cat# ST506) and protease inhibitor cocktail (Sigma-Aldrich, Cat# P8340) on ice for 30 minutes. After centrifuging at 12,000 × rpm for 10 minutes at 4°C, the supernatants were then incubated with anti-Flag, anti-HA, or anti-GFP affinity gel at 4°C for more than 4 h. The gels were subsequently washed three to five times with ice-cold 1× TBS (Sangon Biotech, Cat# B548105) before boiling them with 1× SDS-PAGE loading buffer (YEASEN, Cat# 20315ES) to elute the immune complexes. To analyze the precipitated proteins and the total proteins in the whole cell lysates, SDS-PAGE was performed. The proteins were then transferred onto a PVDF membrane (Millipore, Cat# ISEQ00010), blocked with 5% nonfat dry milk for 2 h at room temperature, hybridized with primary antibodies at 4°C, and finally probed with HRP-conjugated secondary antibodies at room temperature. The labeled bands were visualized using a Tannon 4200 imaging system (Biotanon, China).

### GST affinity-isolation assay

Prokaryotic expression plasmids (GST-tagged IKKε and empty vector) were transformed into BL21 (DE3) competent cells (Vazyme, Cat# C504-03). Once the OD_600_ reached between 0.6 and 0.8, the fresh bacterial solutions were immediately induced with 0.5 ~ 1.0 mM isopropyl-β-D-thiogalactopyranoside (Beyotime, Cat# ST098) for 16 h at 16°C. The recombinant proteins were purified using GST Protein Interaction Pull-Down Kit (Beyotime, Cat# P2250) according to the protocols recommended by the manufacturer. The prey protein (GETV-nsP2) and bait proteins (GST-tagged IKKε and empty vector) purified by GST glutathione agarose resin were incubated together at 4°C. Then, the proteins were washed three times with precooled phosphate-buffered saline (PBS) containing 1% Triton X-100 (Beyotime, Cat# ST795), followed by PBS. Finally, western blotting was performed to determine the protein interactions.

### Indirect immunofluorescence assay

PK15 or HeLa cells were seeded on confocal dishes, fixed with 4% paraformaldehyde (Beyotime, Cat# P0099) for 15 minutes, permeabilized with 0.1% Triton X-100 (Beyotime, Cat# P0096) for 10 minutes, and then blocked with 5% bovine serum albumin (YEASEN, Cat# 36101ES50) in PBS for 1 h. The cells were then separately incubated with the indicated primary antibodies, followed by fluorescently labeled secondary antibodies. Nuclei were stained with 4′,6-diamidino-2-phenylindole (Beyotime, Cat# C1006) for 5 minutes at room temperature. A confocal laser scanning microscope was used to visualize fluorescence in cells, and fluorescent images were taken with the LAS AF Lite software v2.3.5.

### Sequence alignment and structure modeling

Multiple sequence alignment was performed using MEGA-X software, and the figure was prepared with ESPript v3.0 (73). The secondary structure of the GETV nsP2 sequence was determined according to previous studies (13, 74). The three-dimensional structures of this sequence were modeled using AlphaFold2 (75) and visualized by the open-source program PyMOL v2.5.2 (76).

### Statistical analysis

All data were processed and analyzed in Excel and GraphPad Prism v8. Data are presented as means ± standard deviations. Significance was evaluated using a Student’s two-tailed unpaired *t* test to analyze the differences in multiple groups (≥3). Statistical significance was set at *P* < 0.05, 0.01, or 0.001 (*, *P* < 0.05; **, *P* < 0.01; ***, *P* < 0.001; and ns, no significance).

## SUPPLEMENTAL MATERIAL

Supplemental figures and tables spectrum.03419-25-s0001.pdf Fig. S1 to S4, and Tables S1 and S2.

ASM does not own the copyrights to Supplemental Material that may be linked to, or accessed through, an article. The authors have granted ASM a non-exclusive, world-wide license to publish the Supplemental Material files. Please contact the corresponding author directly for reuse.
