# Supplementary material for: Getah virus non-structural protein 2 inhibits type I interferon production by preventing K63-linked polyubiquitination of IKKε and causing widespread cellular shutoff
Source: Microbiol Spectr. 2026 Jun 4;14(7):e03419-25. doi: 10.1128/spectrum.03419-25 (PMC13340153; doi:10.1128/spectrum.03419-25)
Supplement: Supplemental figures and tables — Fig. S1 to S4, and Tables S1 and S2. [file spectrum.03419-25-s0001.pdf]

Supplementary Figures

Supplementary Fig. S1

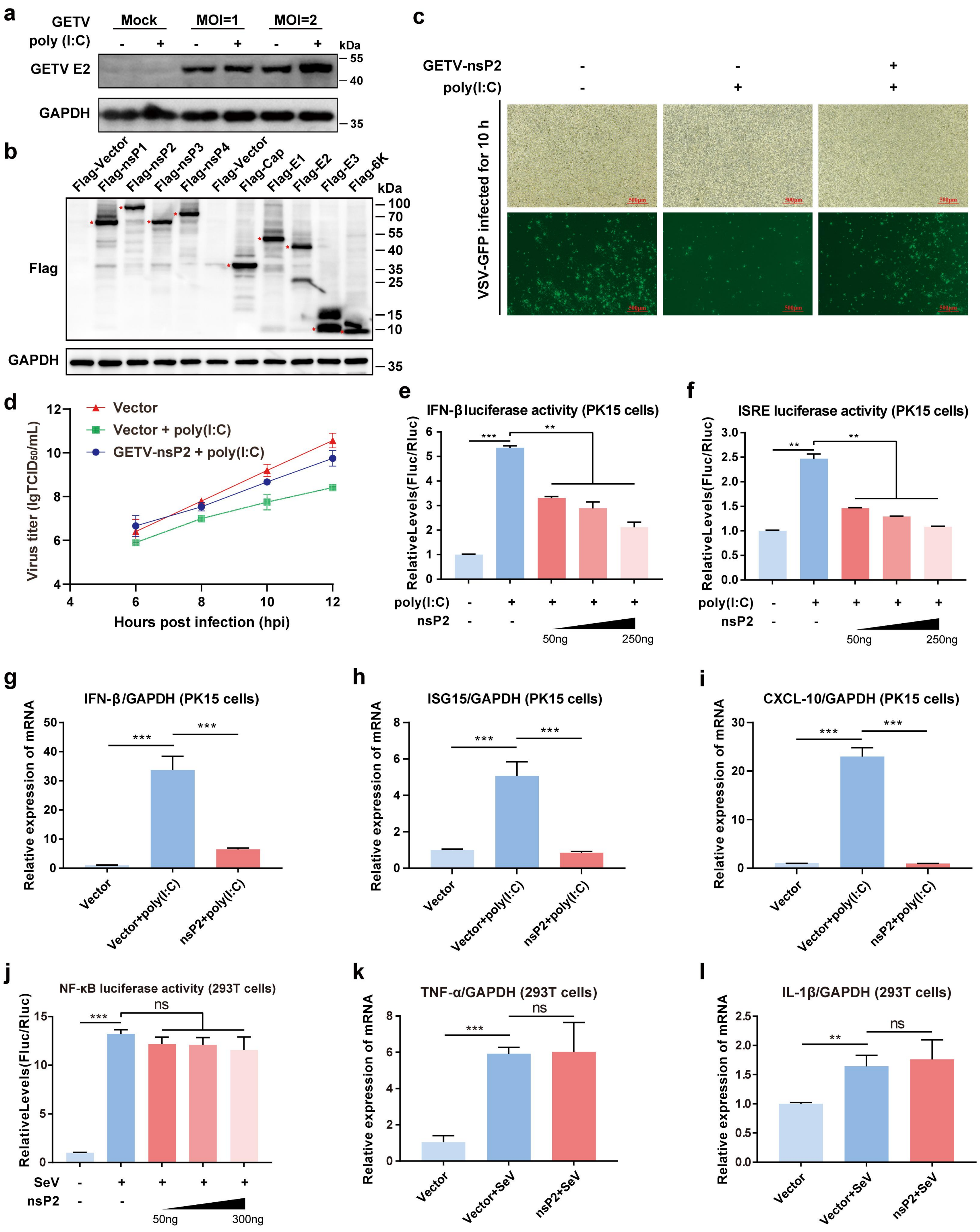

Supplementary Fig. S1 | GETV nsP2 protein inhibits IFN-I production. (a) PK15 cells were transfected with the pGL3-IFN-β-Lucreporter plasmid together with pRL-TK-Luc control plasmid, then were mock-infected or infected with GETV at a MOI of 0.5 and 1 for 24 h. At 12 h post-infection, the cells were transfected with poly(I:C) or left untreated for an additional 12 h, and the cell lysates were subjected to western blotting using anti-GETV E2 polyclonal antibody. GAPDH was used as a protein loading control. **(b)** HEK293T cells were transfected with empty vector or plasmids expressing the indicated viral proteins. After 36 h post-transfection, cells were harvested for western blotting analysis. Expression levels of different GETV proteins were measured by anti-Flag antibody, with GAPDH as an internal loading control. **(c)** HEK293 cells were transfected with the indicated plasmids for 24 h, then stimulated with poly(I:C) for 12 h. The supernatants were harvested to treat fresh HEK293 cells for another 24 h. The cells were then infected with VSV-GFP (MOI = 0.1) for an another 10 h, and GFP expression was observed by the fluorescence microscopy. Scale bar, 500 µm. **(d)** Supernatants from **(c)** were collected and subjected to $TCID_{50}$ assay to measure the titers of the released VSV-GFP progeny virus. **(e, f)** PK15 cells were transfected with increasing amounts of GETV nsP2 expression plasmids or empty vector, along with pGL3-IFN-β-Luc **(e)** or pGL3-ISRE-Luc **(f)** and pRL-TK-Luc plasmids. At 24 h post-transfection, cells were left untreated or treated with poly(I:C) for an additional 12 h, and then luciferase activities were measured. **(g-i)** Quantitative PCR analysis of IFN-β **(g)**, ISG15 **(h)**, and CXCL-10 **(i)** mRNA levels in PK15 cells transfected with GETV nsP2 expression plasmid or empty vector. At 24 h post-transfection, cells were left untreated or treated with poly(I:C) for an additional 12 h. GAPDH was used as an internal control gene for normalization. **(j)** HEK293T cells were transfected with increasing amounts of nsP2-expressing plasmids or empty vector, along with pGL3-NF-κB-Luc and pRL-TK-Luc plasmids. After 24 h of transfection, the cells were infected (or not) with SeV for an additional 12 h, and then luciferase activities were measured. **(k, l)** Quantitative PCR analysis of TNF-α **(k)** and IL-1β **(l)** mRNA levels. All data are representative of triplicates, means ± SD. **, $P < 0.01$; ***, $P < 0.001$ upon Student's t test (two-tailed). MOI, multiplicity of infection.

Supplementary Fig. S2

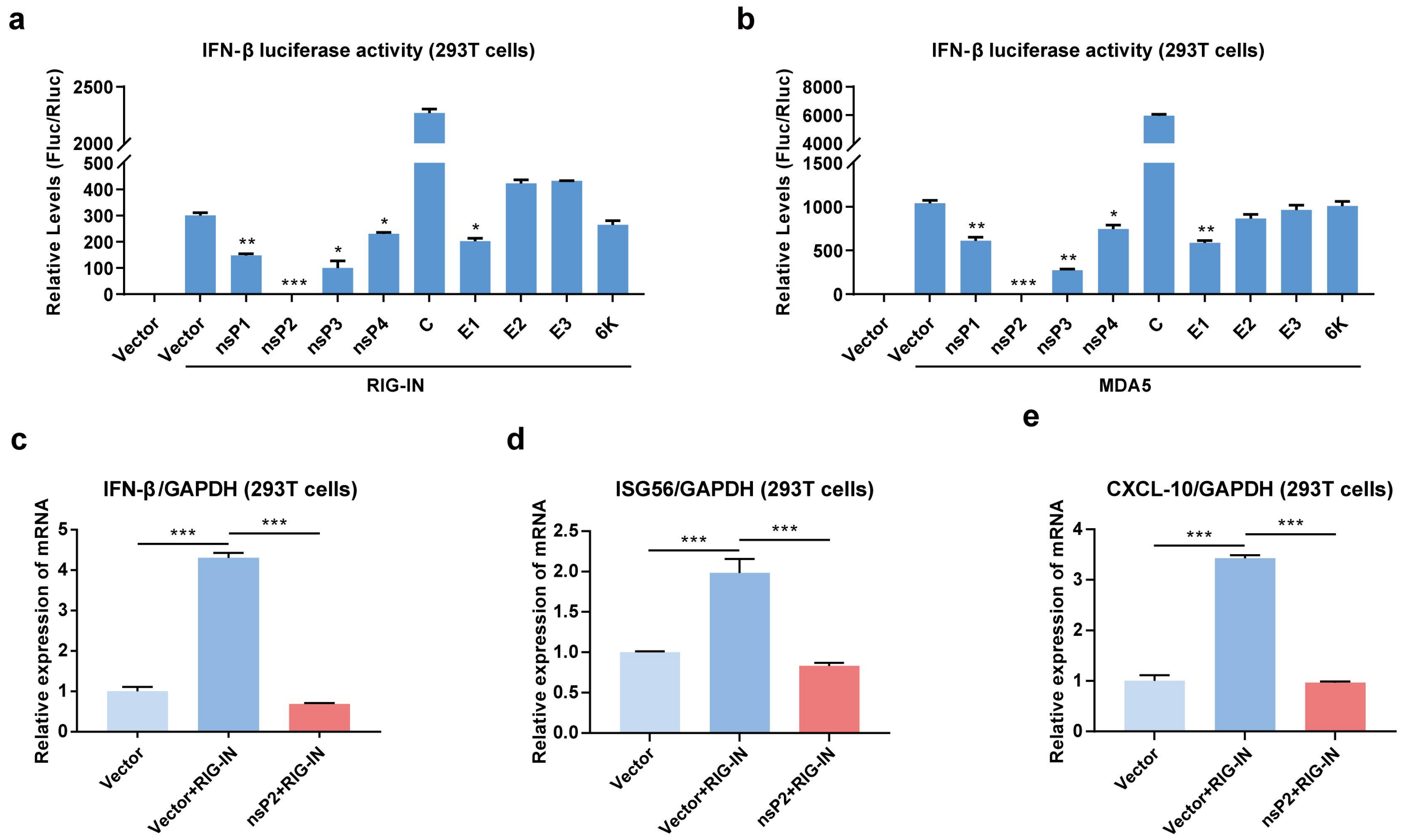

Supplementary Fig. S2 | GETV nsP2 inhibits IFN-β activation by affecting the RLR signaling pathway. (a, b) Effects of nine GETV proteins on RIG-IN **(a)** or MDA5-induced **(b)** IFN-β promoter activation. HEK293T cells were transfected with the pGL3-IFN-β-Luc reporter plasmid, pRL-TK-Luc control plasmid, or plasmids expressing the indicated viral proteins, together with the RIG-IN or MDA5 expression plasmid. After 24 h of transfection, the luciferase activities were measured. **(c-e)** Quantitative PCR analysis of IFN-β **(c)**, ISG56 **(d)**, and CXCL-10 **(e)** mRNA levels in HEK293T cells transfected with empty vector or GETV nsP2 plasmid together with or without RIG-IN-expressing plasmid. GAPDH was used as an internal control gene for normalization. All Data represent means ± SD of triplicate samples, two-tailed Student's *t* test. *, $P < 0.05$; **, $P < 0.01$; ***, $P < 0.001$.

Supplementary Fig. S3

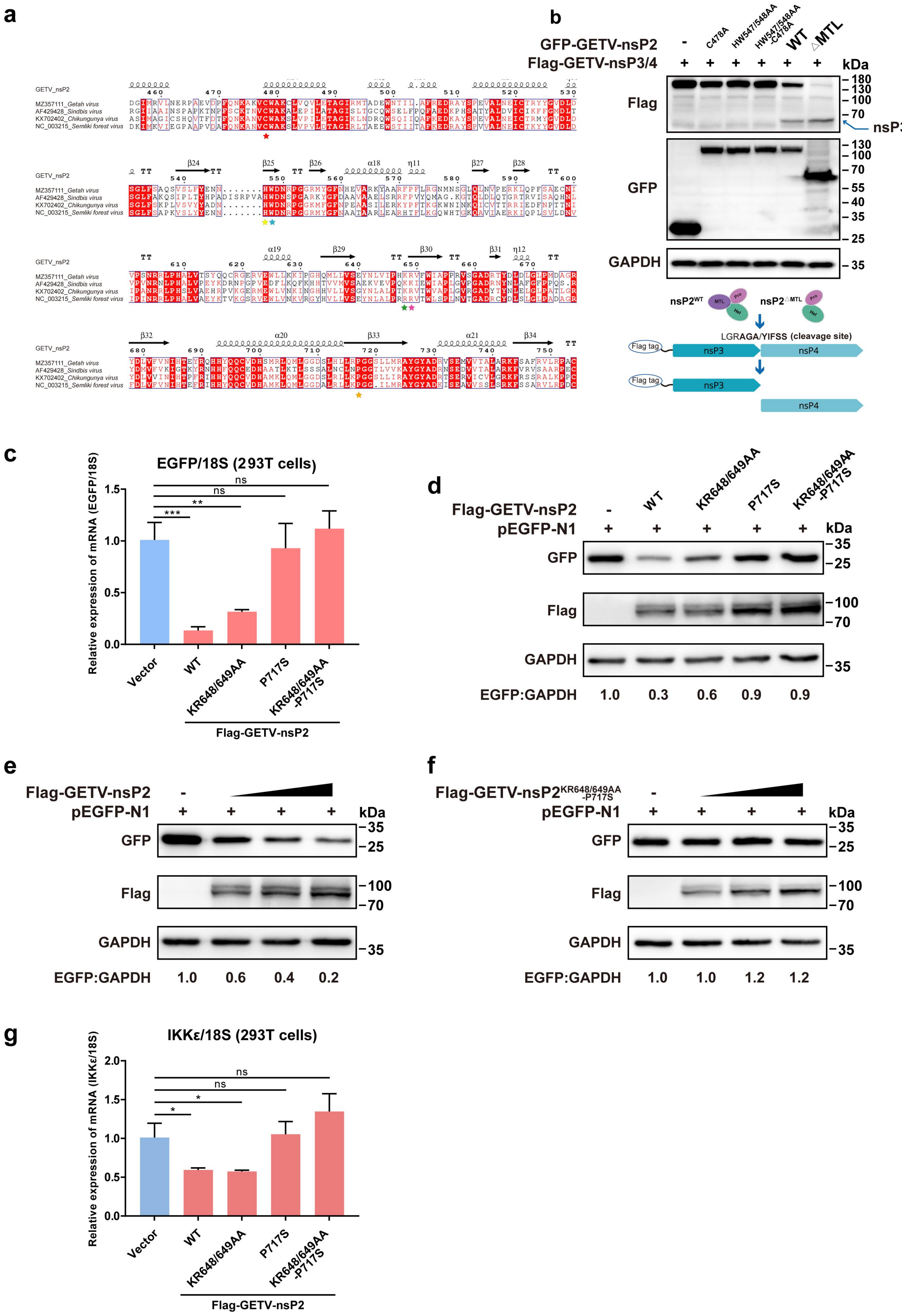

Supplementary Fig. S3 | GETV nsP2 possesses both protease and host shut-off activities. (a) Amino acid alignment of the nsP2 C-terminal region from GETV (GenBank no. MZ357111), SINV (GenBank no. AF429428), CHIKV (GenBank no. KX702402), and SFV (GenBank no. NC_003215). Multiple sequence alignment was performed using MEGA-X software, and the figure was prepared with ESPript3.0. Red shading indicates 100% conserved residues for the four viral protein sequences, and residues labeled in red and framed in blue are conserved >70%. The dots indicate gaps in the sequence. The conserved protease's active site residues along with the Proline residue and NLS-related residues are marked with different colored asterisks. **(b)** HEK293T cells were transfected with either a plasmid expressing the nsP3-nsP4 fusion protein alone, or in combination with GFP-tagged wild-type GETV nsP2, a truncation mutant containing the helicase and protease domains, or its enzymatically inactive mutants. Cleavage of the nsP3-nsP4 fusion protein was assessed by western blotting. **(c)** HEK293T cells were transfected with a pEGFP-N1 vector alone or along with the plasmids expressing wild-type or mutant GETV nsP2 and then harvested for RNA. The mRNA levels of EGFP were measured by qRT-PCR and normalized to 18S rRNA. **(d)** HEK293T cells were transfected with a pEGFP-N1 vector alone or along with the wild-type GETV nsP2 or the indicated mutant expression plasmids and then harvested for protein. EGFP protein abundance was analyzed by western blotting with GAPDH as a protein loading control. **(e, f)** HEK293T cells were transfected with a pEGFP-N1 vector alone or together with increasing concentrations of Flag-tagged wild-type GETV nsP2 **(e)** or its mutant nsP2$^{KR648/649AA\text{-}P717S}$ expression plasmid **(f)**. EGFP and GETV nsP2 protein levels were analyzed by western blotting using anti-GFP and anti-Flag antibodies, respectively, with GAPDH as a protein loading control. **(g)** HEK293T cells were transfected with either empty vector or wild-type nsP2 or the indicated mutant expression plasmids for 36 h and then harvested for RNA. The IKKε mRNA was measured by qRT-PCR and normalized to 18S rRNA. Data represent means ± SD per group. *, $P < 0.05$; **, $P < 0.01$; ***, $P < 0.001$; ns, non-significant upon Student's *t* test (two-tailed).

Supplementary Fig. S4

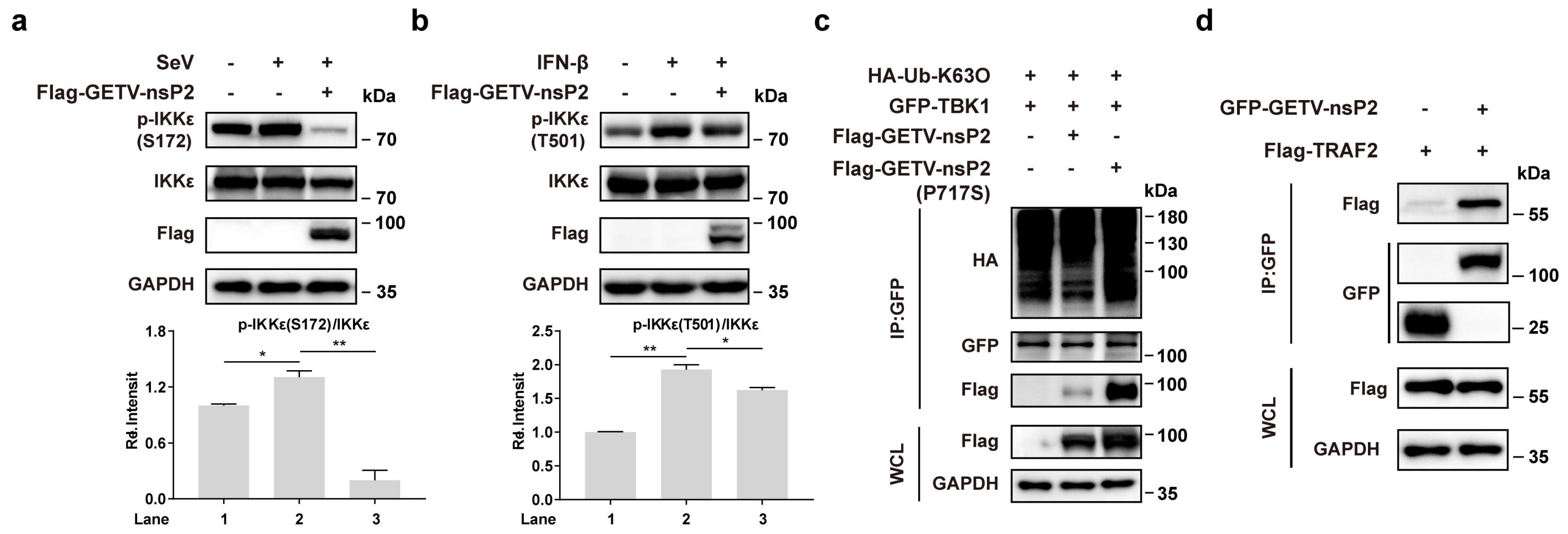

Supplementary Fig. S4 | GETV nsP2 decreases IKKε phosphorylation. **(a)** HEK293T cells were co-transfected with the IKKε-expressing plasmid and Flag-tagged GETV nsP2 plasmid. At 24 h post-transfection, cells were infected (or not) with SeV for an additional 12 h. Cell lysates were then analyzed by western blotting using antibodies against phosphorylated IKKε (Ser172), total IKKε, and GAPDH. **(b)** HEK293T cells were co-transfected with the IKKε-expressing plasmid and Flag-tagged GETV nsP2 plasmid. At 24 h post-transfection, cells were mock-stimulated or stimulated with 10ng/ml IFN-β for 60 min. Cell lysates were then analyzed by western blotting using antibodies against phosphorylated IKKε (Thr501), total IKKε, and GAPDH. **(c)** HEK293T cells were co-transfected with plasmids expressing HA-Ub-K63O, GFP-TBK1, and Flag-tagged wild-type nsP2 or its mutant nsP2^{P717S}. **(d)** HEK293T cells were co-transfected with Flag-TRAF2 and GFP-nsP2 plasmids. After 36 h, cell lysates were subjected to co-immunoprecipitation with GFP-affinity gel, followed by western blotting analysis.

Supplementary Tables

Supplementary Table S1. Primers used for construction of plasmids in this study.

Primer name	Primer sequence (5'-3')
Flag-GETV-nsP1-F	TGAACCGTCAGAATTAAGCTTATGAAGGTAACCGTGGACGTTG
Flag-GETV-nsP1-R	ATCAGATCTATCGATGAATTCTCGGCTCCGGCTCTGAAGGTTA
Flag-GETV-nsP2-F	TGAACCGTCAGAATTAAGCTTATGGGGGTTGTGGAAACACCCAG
Flag-GETV-nsP2-R	ATCAGATCTATCGATGAATTCTCACAACCAGCAGTGTGCAATC
Flag-GETV-nsP3-F	TGAACCGTCAGAATTAAGCTTATGGCACCGTCATACAGGGTCCG
Flag-GETV-nsP3-R	ATCAGATCTATCGATGAATTCTCCGCGCCAGCCCTGCCTAGTC
Flag-GETV-nsP4-F	TGAACCGTCAGAATTAAGCTTATGTATATCTTTTCGTCTGACAC
Flag-GETV-nsP4-R	ATCAGATCTATCGATGAATTCTCTTTAGGACCGCCGTACAGATG
Flag-GETV-C-F	TGAACCGTCAGAATTAAGCTTATGAATTACATTCCAACTC
Flag-GETV-C-R	ATCAGATCTATCGATGAATTCTCCCATTCTTCTGTTCCTTCTG
Flag-GETV-E3-F	TGAACCGTCAGAATTAAGCTTATGTCCGCCGCCTTGATGATGTG
Flag-GETV-E3-R	ATCAGATCTATCGATGAATTCTCGCGACGGTGGCGTGCACTG
Flag-GETV-E2-F	TGAACCGTCAGAATTAAGCTTATGAGTGTGACGGAACACTTCA
Flag-GETV-E2-R	ATCAGATCTATCGATGAATTCTCGGCATGCGCTCGTGGTGCGC
Flag-GETV-6K-F	TGAACCGTCAGAATTAAGCTTATGGCGTCATTTGCGGAATCTA
Flag-GETV-6K-R	ATCAGATCTATCGATGAATTCTCAGATTTTACGACGGGAGTTC
Flag-GETV-E1-F	TGAACCGTCAGAATTAAGCTTATGTACGAACACACCGCGACGATC
Flag-GETV-E1-R	ATCAGATCTATCGATGAATTCTCGCGGCGCATGGTCACACAC
pAcGFP-GETV-nsP2-F	TCAGATCTCGAGCTCAAGCTTCAATGGGGGTTGTGGAAACACCCAG
pAcGFP-GETV-nsP2-R	GGATCCCGGGCCCGCGGTACCTCAACAACCAGCAGTGTGCAATC
pAcGFP-GETV-nsP2Hel-R	GGATCCCGGGCCCGCGGTACCTCACACCTTAGCCTTGTTTTGGA
pAcGFP-GETV-nsP2proDel-F	TCAGATCTCGAGCTCAAGCTTCAATGGTGTGGAAGACACTGTCG
pAcGFP-GETV-nsP2proDel-R	GGATCCCGGGCCCGCGGTACCTCATACAAGTAACATTTGGTG
pAcGFP-GETV-nsP2MTL-F	TCAGATCTCGAGCTCAAGCTTCAATGGTCCCTGAGAGGAAGCTCC
Flag-IKKε-F	CGATGATGACGCCGGATCCATGATGCAGAGCACAGCCAATTAC
Flag-IKKε-R	ATGCATGCTCGAGCGGCCGCTCAGACATCAGGAGGTGCTG
Flag-IKKε-KD-R	ATGCATGCTCGAGCGGCCGCCTATCGCTGCAGGAUGTCACTGG
Flag-IKKε-SDD-F	CGATGATGACGCCGGATCCATGAGCACAGCCATCCCTAAGG
Flag-IKKε-SDD-R	ATGCATGCTCGAGCGGCCGCCTATTCCAGGAGCTTGCTGAGAC
HA-IKKε-F	CATGGAGGCCCGAATTCCGATGCAGAGCACAGCCAATTAC
HA-IKKε-R	GTCTGGATCCCCGCGGCCGCTCAGACATCAGGAGGTGCTG
GST-IKKε-F	TCGGTACCCTCGAGGGATCCATGCAGAGCACAGCCAATTA
GST-IKKε-R	CAAGCTTGAATTCGGATCCTCAGACATCAGGAGGTGCTG
EGFP-GETV-nsP2-F	TCAGATCTCGAGCTCAAGCTTATGGGGGTTGTGGAAACACCCAG
EGFP-GETV-nsP2-R	GGATCCCGGGCCCGCGGTACCTCACAACCAGCAGTGTGCAATC
C478A-F	CAAAACAAGGCTAAGGTGGCCTGGGCAAAATGTCTGGTG
C478A-R	CACCAGACATTTTGCCCAGGCCACCTTAGCCTTGTTTTG
HW547/548AA-F	CTCTTTTATGAGAACAACGCCGCGGACAACAGGCCTGGAGG
HW547/548AA-R	CCTCCAGGCCTGTTGTCCGCGGCGTTGTTCTCATAAAAGAG
KR648/649AA-F	AACCTGGTGATACCTCACGCAGCAGTCTTCTGGATTGCACCT
KR648/649AA-R	AGGTGCAATCCAGAAGACTGCTGCGTGAGGTATCACCAGGTT
P717S-F	TCACTACACCTGCTCAGATCAGGAGGCTCGCTGCTGATG
P717S-R	CATCAGCAGCGAGCCTCCTGATCTGAGCAGGTGTAGTGA
Flag-RIG-I-F	CGATGATGACGCCGGATCCATGACCACCGAGCAGCGA
Flag-RIG-I-R	ATGCATGCTCGAGCGGCCGCTCATTTGGACATTTCTGCTGGA
Flag-MDA5-F	CGATGATGACGCCGGATCCATGTCGAATGGGTATTCCAC
Flag-MDA5-R	ATGCATGCTCGAGCGGCCGCCTAATCCTCATCACTAAATAAAC
Flag-MAVS-F	CGATGATGACGCCGGATCCATGCCGTTTGCTGAAGACAAGA
Flag-MAVS-R	ATGCATGCTCGAGCGGCCGCCTAGTGCAGACGCCGC
Flag-TBK1-F	CGATGATGACGCCGGATCCATGCAGAGCACTTCTAATCATC
Flag-TBK1-R	ATGCATGCTCGAGCGGCCGCCTAAAGACAGTCAACGTTG
Flag-IRF3-F	CGATGATGACGCCGGATCCATGGGAACCCCAAAGCCAC
Flag-IRF3-R	ATGCATGCTCGAGCGGCCGCCTAGCTCTCCCCAGGGCCCT
Myc-TRAF2-F	GAAGAGGATCTGTCGGGTACCATGGCTGCAGCTAGCGTGAC
Myc-TRAF2-R	TGCTGGATATCTGCAGAATTCTTAGAGCCCTGTCAGGTCCA

Supplementary Table S2. Primers used for qRT-PCR in this study.

Gene	Species	Forward primer (5'-3')	Reverse primer (5'-3')
GAPDH	Human	GGAGCGAGATCCCTCCAAAAT	GGCTGTTGTCATACTTCTCATGG
ISG56	Human	TCCTTGGGTTCGTCTACAAAT	TTCTCAAAGTCAGCAGCCAGT
CXCL10	Human	GTGGCATTCAAGGAGTACCTC	GACCTTTCCTTGCTAACTGCT
IFN-β	Human	TTGCTCTCCTGTTGTGCTTC	AAGCCTCCCATTCAATTGCC
IKKε	Human	TCTTTGCGGAGACCAGTGAC	ATCGTGTTGTGGGCATGGAT
TNF-α	Human	CTCTTCTGCCTGCTGCACTTTG	ATGGGCTACAGGCTTGTCACTC
IL-1β	Human	CCACAGACCTTCCAGGAGAATG	GTGCAGTTCAGTGATCGTACAGG
18S rRNA	Human	GTAACCCGTTGAACCCCATT	CCATCCAATCGGTAGTAGCG
GAPDH	Pig	TTTAACTCTGGCAAAGTGGACA	GGCCTTTCCATTGATGACAAGC
ISG15	Pig	GCAGCAACGCCTATGAGGTCT	AGGCTTGAGGTCATACTCCCC
CXCL10	Pig	ACTGTTCGCTGTACCTGCAT	TGATCTCAACATGTGGGCAAG
IFN-β	Pig	CATCCTCCAAATCGCTCTC	TCATCCTATCTTCGAGGCAA
EGFP	-	GAACCGCATCGAGCTGAA	TGCTTGTCGGCCATGATATAG
